# The incidence of outpatient care within 24 months from SARS-CoV-2 infection in the general population: a multicenter population-based cohort study

**DOI:** 10.1186/s12879-025-10526-0

**Published:** 2025-01-30

**Authors:** Federico Banchelli, Carlo Gagliotti, Angela De Paoli, Rossella Buttazzi, Elena Narne, Enrico Ricchizzi, Silvia Pierobon, Ugo Fedeli, Gisella Pitter, Elisa Fabbri, Michele Tonon, Elisa Gentilotti, Maurizia Rolli, Evelina Tacconelli, Maria Luisa Moro, Francesca Russo, Elena Berti

**Affiliations:** 1 Department of innovation in healthcare and social services, Emilia-Romagna Region, Bologna, Italy; 2 Regional Health and Social Care Agency, Emilia-Romagna Region, Bologna, Italy; 3 Azienda Zero, Padova, Italy; 4 Directorate of prevention, food safety, and veterinary public health, Veneto Region, Venezia, Italy; 5 Department of Diagnostics and Public Health, University of Verona, Verona, Italy

**Keywords:** SARS-CoV-2, Post-COVID, Long-term COVID-19 sequelae, Outpatient care, Pre-post study, Population-based cohort, ORCHESTRA project

## Abstract

**Background:**

The long-term effects of COVID-19, which can vary significantly in type and timing, are considered relevant and impacting on the well-being of individuals. The present study aims to assess the incidence of outpatient care in the post-acute phase of SARS-CoV-2 infection in two Italian regions.

**Methods:**

The study has a multicentre, population-based, pre-post, repeated measures design to compare the incidence rate of access to outpatient visits and diagnostics before and after SARS-CoV-2 infection, considering a follow-up of 24 months. The study made use of previously recorded large-scale healthcare data available in the administrative databases of the Emilia-Romagna (E-R) and Veneto regions. Analyses were carried out separately in the two regions and results were pooled using random effects meta-analysis.

**Results:**

There were 27,140 subjects in E-R and 22,876 in Veneto who were included in the analysis. The pooled outputs showed an increase in rates of outpatient visits and diagnostics starting from month 2 after SARS-CoV-2 infection (IRR = 1.68, 95% CI = 1.56–1.81) with a peak at month 4 (IRR = 2.05, 95% CI = 1.95–2.15); the increase continued with reduced intensity up to month 15. Stratified analysis revealed that subjects with severe acute COVID-19 had a higher increase in rates (up to IRR = 3.96, 95% CI = 2.89–5.44), as well as patients with no comorbidities (up to IRR = 2.71, 95% CI = 2.60–2.83).

**Conclusion:**

Long-term effects of COVID-19 include an increase in the healthcare burden especially in the first months after the acute infection. The increased demand for resources can last up to two years after infection in particular subgroups of patients such as subjects admitted to hospital during the acute phase due to the severe presentation of the disease.

**Supplementary Information:**

The online version contains supplementary material available at 10.1186/s12879-025-10526-0.

## Introduction

COVID-19 can cause a wide range of long-term outcomes that include various sequelae (psychiatric sequelae, thrombosis, intubation-related trauma, etc.) and a multisystem condition following an acute SARS-CoV-2 infection known as long COVID [1–5]. The long-term effects of COVID-19 may vary widely in type and timing, as they can follow initial recovery or persist from the acute episode. Symptoms may also fluctuate, relapse, or change over time [1–5]. Based on a conservative occurrence of this condition in 10% of infected persons, it is estimated that there are many millions of cases worldwide [1, 6]. The incidence varies according to the severity of the acute infection and age [1, 7]. It is also higher in hospitalized patients [1]. Considering, however, that non-hospitalized cases are the great majority, the absolute number of long-term effect of COVID-19 is higher in this category of patients [1]. There are still many open questions about the pathophysiology, risk factors and management of long-term effects of COVID-19 [6]. These effects encompass multiple adverse outcomes including cardiovascular, thrombotic, cerebrovascular, endocrine (type 2 diabetes), and neurological (myalgic encephalomyelitis/chronic fatigue syndrome) diseases, and can lead to a lower quality of life and to an increased need for access to specific healthcare services [1, 3, 8–13]. The long-term effects of COVID-19 are described for variable durations, estimated to last even for life, and can be influenced by various pre-existing risk factors and patient characteristics such as age, female sex, ethnicity (1, 3, 8–9, 13, 14, 15, 16, 17, 18, 19, 20, 21, 22, 23). Evidence in literature also suggests the possibility that the symptoms of long COVID might have a cluster distribution [24].

A previous study highlighted a remarkable use of outpatient care in the post-acute phase, in a population with history of SARS-CoV-2 infection and at low risk of severe acute disease in the E-R and Veneto regions [25]. The present study extends those findings by including all individuals with SARS-CoV-2 infection, regardless of risk of severe disease, and by prolonging the follow up from 12 to 24 months. To understand the occurrence and duration of healthcare utilization that may be related to long-term effects of COVID-19 in a large population would be of great importance to guide clinical management and allocation of appropriate resources.

## Methods

The study was carried out within the context of the EU’s Horizon 2020 research project called ORCHESTRA (Connecting European Cohorts to increase common and effective response to SARS-CoV-2 pandemic) (www.orchestra-cohort.eu).

### Data source

The study was carried out in Emilia-Romagna (E-R) and Veneto, two neighbouring regions of northern Italy with a population of approximately 4.4 and 4.9 million residents, respectively. Both these two regions oversee a regional healthcare system and have exclusive competence in regulating, financing, and organizing healthcare services and activities carried out within their territory. Data were extracted from the E-R and Veneto regions healthcare administrative databases, which include comprehensive information about healthcare provision by the regional healthcare systems. Secure record-linkage procedures were carried out at the individual level to merge pseudonymized data related to official notifications of SARS-CoV-2 infections, outpatient care, residence and vital status, acute hospital admissions, and community hospital admissions. Data sources have been standardised between the two regions by using common rules for the definition of variables.

### Study aim

The aim of the present study was to assess the incidence of access to specific outpatient care visits and procedures within 24 months from confirmed SARS-CoV-2 infection, compared to the pre-infection period, in the general population of E-R and Veneto regions in Italy.

### Study population

The population of interest included all consecutive adults with a first confirmed SARS-CoV-2 infection episode (molecular or antigen tests) in the E-R and Veneto regions between February 2020 and September 2020 who were alive after the acute phase of the disease. Eligible individuals included all subjects aged ≥ 18 years at the time of SARS-CoV-2 diagnosis and with continuous residence status in the E-R or Veneto regions in the year prior to diagnosis. None of these subjects was vaccinated at the time of SARS-CoV-2 diagnosis, as the vaccination campaign in Italy only started in late December 2020.

### Study design

The study uses a multicentre, population-based, pre-post, repeated measures design to compare the incidence rate of access to specific healthcare services before and after SARS-CoV-2 infection. This quasi-experimental pre-post study design was used to assess the fraction of outcomes attributable to COVID-19, by comparing outcomes that occurred before and after SARS-CoV-2 infection in the same individuals [26]. Using this study design, each individual is compared with her/himself and the difference between the outcomes occurred after and before the infection can be attributed to COVID-19. The risk of history bias related to external events occurring through the study period was controlled for within the statistical analysis. In this study, two sources of history bias were considered: ageing of individuals, which can contribute to the development of new diseases or worsening of previous conditions, and differences in levels of provision of healthcare services due to lockdowns and other restriction measures, seasonality, and cyclical events (e.g., summer holidays). Individuals entered the cohort on the day of SARS-CoV-2 diagnosis and were assessed within one year before (control period: CP) and two years after the diagnosis. Outcomes measured in the 30 days period before the infection were excluded from the analysis, to avoid misclassification of events with respect to the timing of the infection, which may have occurred before the molecular or antigen test. We distinguished an acute phase (AP) from diagnosis till day 30 and a post-acute phase (PAP) from day 31 till 2 years after diagnosis. The outcome was assessed repeatedly for each individual in several periods of 30 days each. Based on these repeated measures, the outcome incidence rate in each month of the PAP was compared with that observed in the CP. Outcomes occurred during the AP were not considered. Individuals who died, moved residence out of the E-R or Veneto regions, or had a SARS-CoV-2 reinfection during the PAP exited the cohort on the date of the first of these events. In each 30-days period, we calculated the number of outpatients care services of interest and the time at risk. The time at risk was defined as the number of days the subject was alive, resident in the E-R or Veneto regions and not hospitalized. Individuals who died or moved out of the regions during the AP, or who did not have at least one day at risk in the CP and in the PAP, were excluded from the analysis. Duplicate or incomplete records were discarded. The study was carried out and reported according to the GATHER statement [27].

### Study outcome

In this study, we used an approach based on health databases as a source of information, considering access to care as a proxy for the long-term effects of COVID-19 requiring medical attention, including both newly occurring conditions and worsening of pre-existing ones [28]. The outcome of interest was the access to specific healthcare services during the PAP, defined through a selected list of outpatient care visits and procedures. Selected outpatient care included ambulatory visits in cardiology, pneumology, angiology, neurology, psychiatry, rehabilitation-motor, nephrology and diabetes, as well as other diagnostic and therapeutic procedures such as chest imaging, cardiac ultrasound imaging, pneumological diagnostics, electrocardiography, oxygen therapy, respiratory and cardiological rehabilitation, peripheral vascular ultrasound imaging, training for cognitive disorders, haemodialysis, renal imaging, and glycated haemoglobin analysis [25, 28–30]. Detailed extraction criteria for these outpatient care services, based on regional outpatient codes, has been described in a previous study [25].

### COVID-19 severity

The primary analyses were stratified by acute COVID-19 severity. COVID-19 severity was assigned algorithmically based on respiratory system diagnoses (i.e. acute respiratory insufficiency, pneumonia, acute lower respiratory tract infections, other respiratory diagnoses), on ventilation procedures administered (i.e. oxygen therapy, non-invasive ventilation, invasive ventilation), and on intensive or sub-intensive care unit stay during hospitalizations in the AP. It is expressed on a four-level ordinal scale as low, mild, moderate, or severe. For subjects not hospitalized in the AP, the “low” level of severity was assigned. The COVID-19 severity algorithm has been described in a previous study [25].

### Comorbidities

The seventeen conditions considered by the Charlson comorbidity index were reported, based on previously published criteria [31]. Comorbidities were defined based on hospital discharge records that occurred within five years before the infection. A supplementary analysis was stratified by number of Charlson comorbidities: no comorbidities; one comorbidity; more than one comorbidity.

### Statistical analysis

Statistical analysis was carried out separately in the two regions, following a common analysis plan, and the main results were pooled using meta-analysis. The frequency distributions of the categorical characteristics were described as numbers and percentages, whereas numerical variables were described as the mean ± standard deviation. The observed crude incidence rates of outpatient care were calculated as the number of outpatient care services per 1,000 individuals per day at risk. The comparison of incidence rates in the PAP and in the CP was carried out using a repeated measures generalized Poisson mixed model [32]. The dependent variable was the number of selected outpatient care services, whereas the independent variables were: 23 dummy variables, one for each month of the PAP and considering the CP as the reference; the time at risk in the 30-day period (as an offset variable); the age of the individual at the beginning of the 30-day period; and the average provision level of outpatient care in the 30-day period. The average provision level in each 30-day period is the observed daily average total number of outpatient care services in that period in the Local Health Unit of residence, normalized for its observed daily average in 2019 (last year before the pandemic) (Supplementary Fig. 1, Additional File 1). The latter two independent variables were included in the model to avoid history bias. Associations were measured using the incidence rate ratio (IRR) and the uncertainty in results was expressed with 95% confidence intervals (CI). More details on the statistical analysis are reported in Additional Documentation (Additional File 1).

## Results

**Fig. 1:**
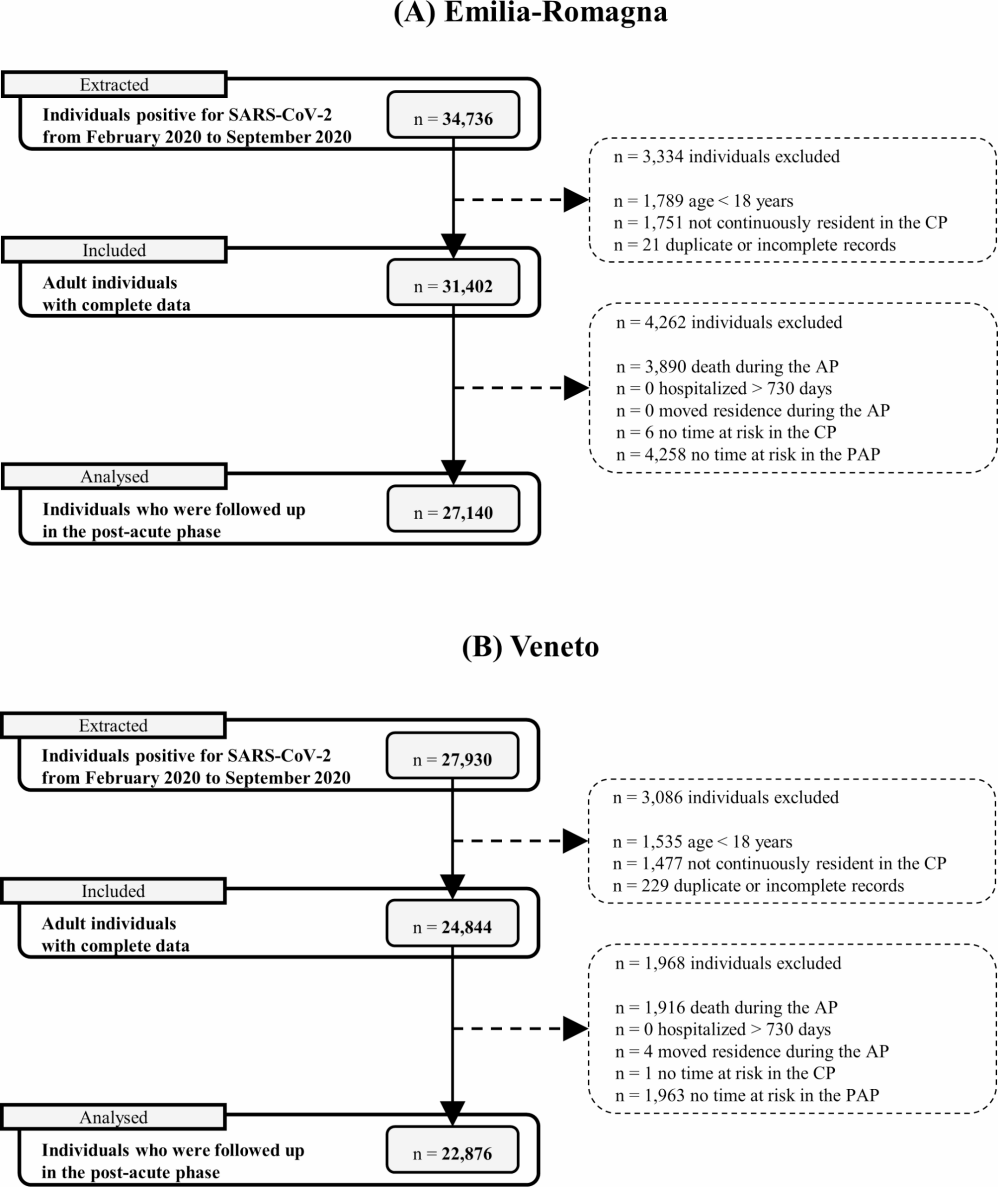
Flow-chart describing inclusion of individuals in the study. Notes: (**A**) = Emilia-Romagna Region; (**B**) = Veneto Region; CP = control period before the SARS-CoV-2 infection; AP = acute phase after the infection; PAP = post-acute phase after the infection

From February 2020 to September 2020, there were 34,736 individuals positive for SARS-CoV-2 in the E-R region and 27,930 in the Veneto region. Individuals who were included in the data analysis numbered 50,016, of which 27,140 in Emilia-Romagna and 22,876 in Veneto (Fig. 1). Males were 46.7% in E-R and 45.6% in Veneto, and those aged ≥ 60 years were 40.7% and 37.3%, respectively. In the Veneto cohort there were fewer individuals who experienced severe (8.3%) or moderate (8.7%) acute COVID-19 than in the E-R cohort (16.2% and 10.8%). Based on the criteria of the Charlson index, 16.5% and 14.0% of subjects have at least one comorbidity and 8.2% and 5.9% have more than one, in E-R and Veneto respectively (Table 1). Descriptive statistics, stratified by acute COVID-19 severity, are reported in Supplementary Table 1 (Additional File 1). The prevalence of each single comorbidity of the Charlson index is reported in Supplementary Table 2 (Additional File 1). The most common ones were cerebrovascular diseases (4.9% in E-R and 3.8% in Veneto), cancer (3.7% and 3.1%), congestive heart failure (3.4% and 3.4%), diabetes without complications (3.7% and 2.6%), and dementia (3.5% and 2.4%).

**Table 1:** Characteristics of the analysed individuals

		Emilia-Romagna (*N* = 27,140)		Veneto (*N* = 22,876)	
		*n*	%	*n*	%
Sex	Male	12,674	46.7%	10,441	45.6%
Age at diagnosis	18–39	5,992	22.1%	5,710	25.0%
	40–49	4,614	17.0%	3,833	16.8%
	50–59	5,496	20.3%	4,813	21.0%
	60–69	3,700	13.6%	2,741	12.0%
	70–79	3,172	11.7%	2,104	9.2%
	≥ 80	4,166	15.4%	3,675	16.1%
Citizenship	Italian	23,998	88.4%	20,084	87.8%
	HMPC	3,096	11.4%	2,756	12.1%
	LMPC	46	0.2%	36	0.2%
Severity of acute COVID-19	Low	19,393	71.5%	18,833	82.3%
	Mild	424	1.6%	143	0.6%
	Moderate	2,925	10.8%	1,997	8.7%
	Severe	4,398	16.2%	1,903	8.3%
Charlson comorbidities	No	22,653	83.5%	19,675	86.0%
	One	2,261	8.3%	1,844	8.1%
	More than one	2,226	8.2%	1,357	5.9%

Notes: HMPC = high migratory pressure countries; LMPC = low migratory pressure countries

The average days at risk in the E-R cohort were 327.7 in the CP and 644.6 in the PAP, whereas in the Veneto cohort they were 328.1 and 634.3. The number of individuals who completed the 24-month follow-up without being censored was 83.8% in E-R and 76.4% in Veneto (Table 2). The same information, stratified by acute COVID-19 severity, is reported in Supplementary Table 3 (Additional File 1). The causes of censoring were similar in both cohorts. Deaths during the PAP occurred in 6.4% of analysed individuals in E-R and in 7.3% in Veneto. SARS-CoV-2 reinfection occurred in 9.3% of individuals in E-R and in 15.6% in Veneto. Only a few subjects had censored times due to moving residence out of the region (0.5% in E-R and 0.7% in Veneto) (Table 2).

**Table 2:** Time at risk and causes of interruption of follow-up

	Emilia-Romagna (*N* = 27,140)	Veneto (*N* = 22,876)
Days at risk in the CP (mean ± SD)	327.7 ± 11.9	328.1 ± 10.3
Days at risk in the PAP (mean ± SD)	644.6 ± 131.4	634.3 ± 141.8
Complete 24-months follow-up (n %)	22,750 (83.8%)	17,485 (76.4%)
Death (n %)	1,736 (6.4%)	1,662 (7.3%)
Moved residence outside the region (n %)	149 (0.5%)	155 (0.7%)
Reinfection (n %)	2,517 (9.3%)	3,574 (15.6%)

Notes: SD = standard deviation; CP = control period before SARS-CoV-2 infection; PAP = post-acute phase after SARS-CoV-2 infection

### Incidence of outcomes

**Fig. 2:**
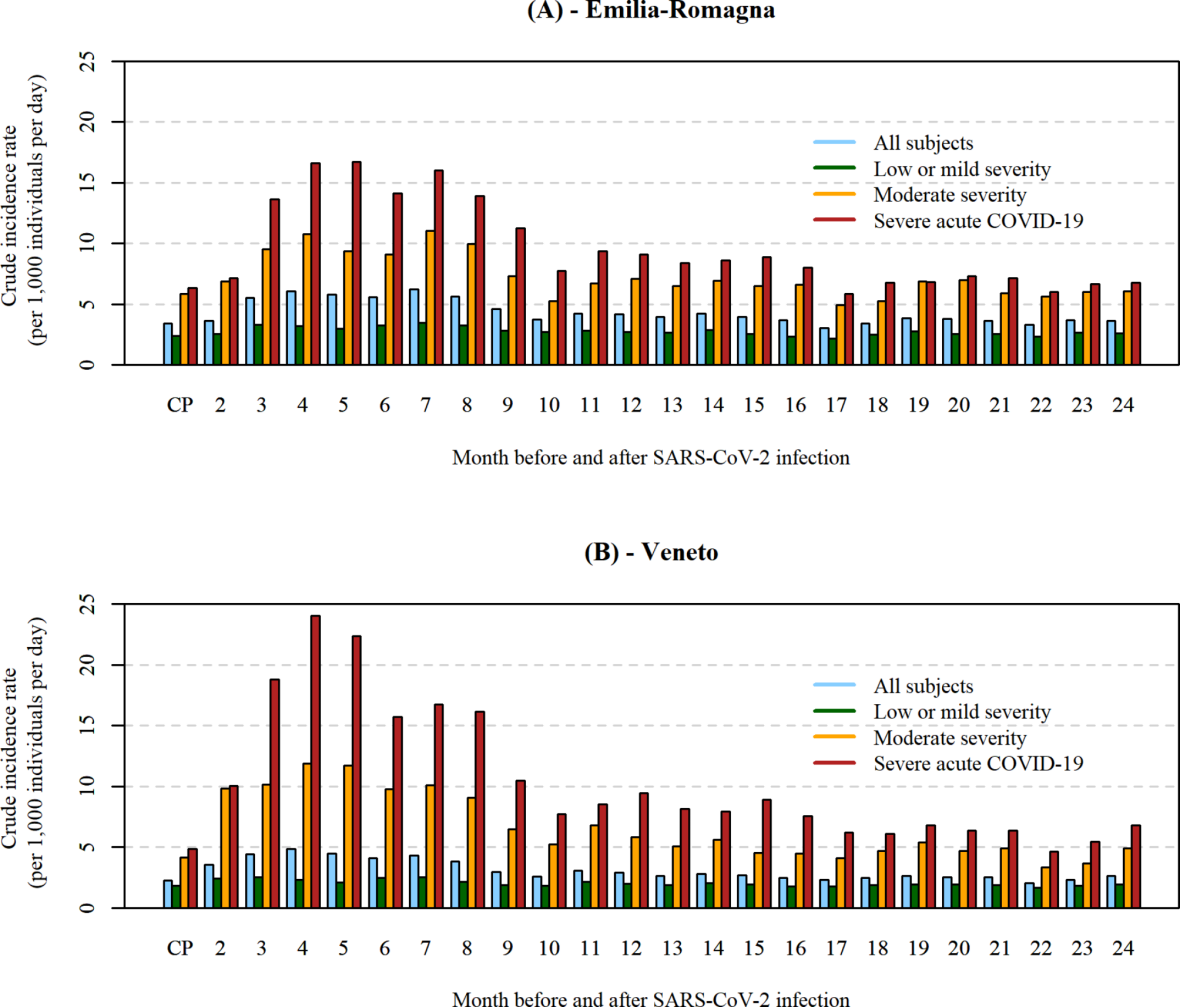
Observed incidence rates of selected outpatient care services before and after SARS-CoV-2 infection, by COVID-19 severity. Notes: Observed crude incidence rates, expressed as the number of outpatient care services per 1,000 individuals per day, are shown for the control period (CP) and for each month of the post-acute phase after SARS-CoV-2 infection; (**A**) = Emilia-Romagna Region; (**B**) = Veneto Region; blue bars represent all analyzed subjects; green bars represent subjects with low or mild COVID-19 severity; yellow bars represent subjects with moderate COVID-19 severity; red bars represent subjects with severe COVID-19

The crude outcome incidence rates in the CP were 3.39 and 2.26 outpatient care services per 1,000 individuals per day, in the general populations of E-R and Veneto (Fig. 2). The rates in the CP were lower among subjects with low severity (2.38 in E-R and 1.81 in Veneto) than among patients with moderate severity (5.83 and 4.16) or of severe patients (6.32 and 4.84). After the acute phase of the infection, rates in the general population rose and peaked at 6.23 outpatient care services per 1,000 individuals per day in E-R (at month 7 after the infection) and at 4.83 in Veneto (at month 4 after the infection). For patients who experienced severe acute COVID-19, the increase in rates was much higher in the first months after the infection, peaking at 16.70 outpatient care services per 1,000 individuals per day in E-R (at month 5) and at 24.05 in Veneto (at month 4). After these peaks, there was a slow but steady decline of rates, which in the general population were equal to 4.18 at month 12 and 3.64 at month 24 after the infection in E-R, and to 2.91 and 2.60 in Veneto, respectively (Fig. 2). For patients without comorbidities, the incidence rate in the CP was 1.87 in E-R and 1.57 in Veneto, much lower than in those with one comorbidity (6.96 and 5.23) as well as more than one comorbidity (15.78 and 8.69). The relative increase in rates was however higher among those with no comorbidities (reaching 4.55 in E-R at 4.30 in Veneto, both at month 3 after the infection), and lower among those with only one comorbidity (reaching 9.21 at month 6 in E-R and at 7.10 at month 2 in Veneto) or more than one comorbidity (reaching 20.87 at month 3 in E-R and at 10.61 at month 6 in Veneto). Detailed incidence data in each cohort are reported in Supplementary Tables 4–11 and Supplementary Fig. 2 (Additional File 1).

### Comparison of rates before and after SARS-CoV-2 infection

**Fig. 3:**
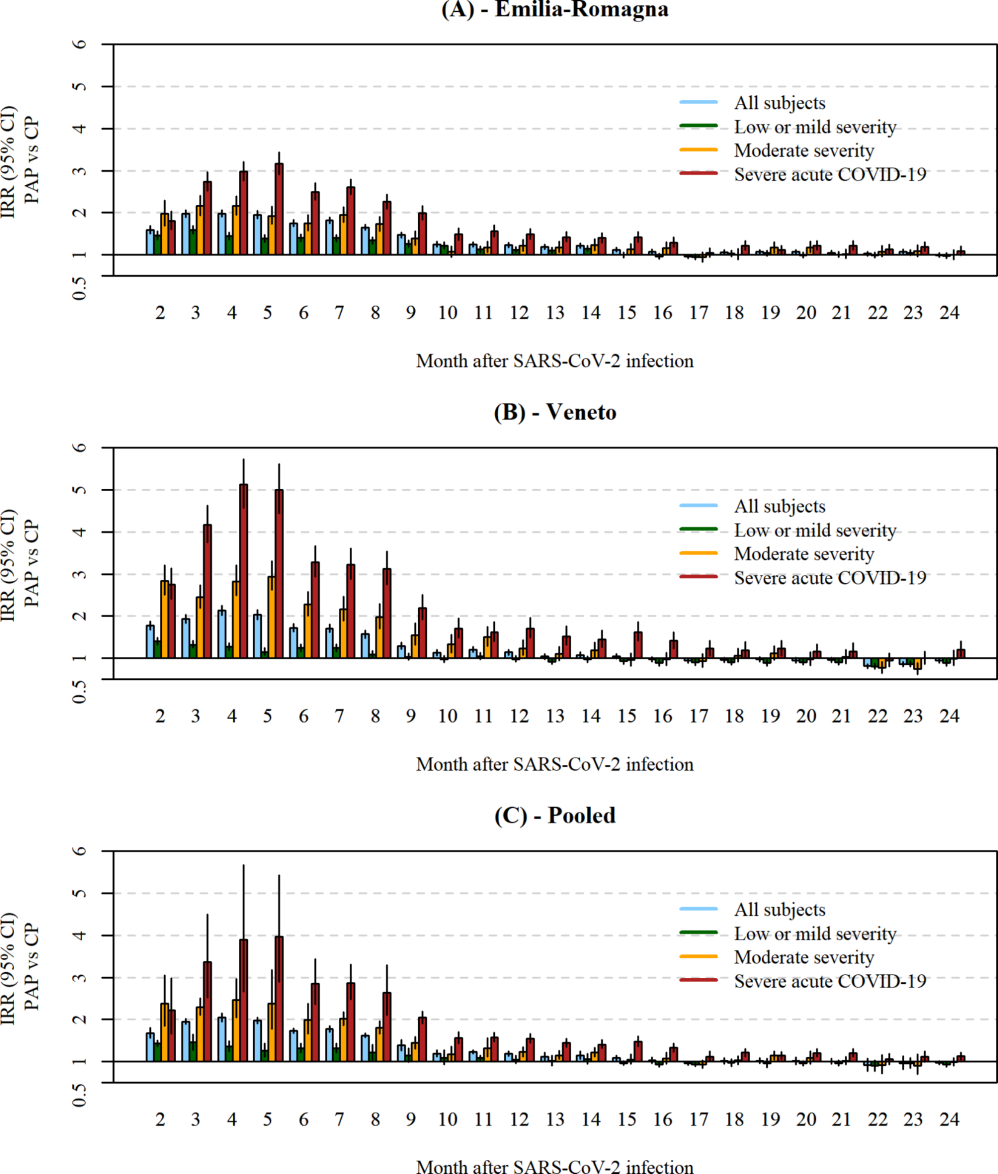
Incidence rate ratio of selected outpatient care services comparing pre- and post-infection periods, by COVID-19 severity. Notes: Incidence rate ratios (IRR) with 95% confidence intervals (CI) are shown for each month of the post-acute phase (PAP) after SARS-CoV-2 infection, compared to the pre-infection control period (CP); (**A**) = Emilia-Romagna Region; (**B**) = Veneto Region; (**C**) = Pooled results; blue bars represent all analyzed subjects; green bars represent subjects with low or mild COVID-19 severity; yellow bars represent subjects with moderate COVID-19 severity; red bars represent subjects with severe COVID-19; vertical error bars represent 95% CI

The comparison of incidence rates of selected outpatient care services before and after SARS-CoV-2 infection, adjusted for history bias, is reported in Fig. 3. In the general population, according to the pooled analysis, there was an increase in rates starting from month 2 after SARS-CoV-2 infection (IRR = 1.68, 95% CI = 1.56–1.81). This increase peaked at month 4 (IRR = 2.05, 95% CI = 1.95–2.15) and continued with decreasing intensity at month 6 (IRR = 1.74, 95% CI = 1.68–1.80), at month 12 (IRR = 1.19, 95% CI = 1.13–1.25) and up to month 15 (IRR = 1.09, 95% CI = 1.03–1.14). In the subgroup of individuals with low or mild severity acute COVID-19 (73.1% of the total population in E-R and 82.9% in Veneto), the increase was less intense and prolonged. The peak was at months 2 (IRR = 1.43, 95% CI = 1.37–1.50) and 3 (IRR = 1.46, 95% CI = 1.28–1.66) and the increase was observed until month 8 (IRR = 1.21, 95% CI = 1.05–1.40). In the subgroup of individuals with moderate severity acute COVID-19 (10.8% of the total population in E-R and 8.7% in Veneto), the peak was higher (more than two-fold increase) and occurred at months 2 (IRR = 2.38, 95% CI = 1.85–3.06) and 5 (IRR = 2.38, 95% CI = 1.77–3.19). The increase persisted continuously up to month 14 (IRR = 1.22, 95% CI = 1.11–1.33). In the subgroup of individuals with severe acute COVID-19 (16.2% of the total population in E-R and 8.3% in Veneto), the increase in rates was remarkably high and prolonged after the infection. The increase was more than two-fold at month 2 after the infection (IRR = 2.23, 95% CI = 1.66–2.98), and almost four-fold at its peak at months 4 (IRR = 3.89, 95% CI = 2.67–5.67) and 5 (IRR = 3.96, 95% CI = 2.89–5.44). Thereafter, the increase declined progressively at month 9 (IRR = 2.05, 95% CI = 1.91–2.19), month 12 (IRR = 1.55, 95% CI = 1.44–1.67), month 18 (IRR = 1.21, 95% CI = 1.12–1.31), and is still present at month 24 (IRR = 1.13, 95% CI = 1.04–1.22), although not continuously and with reduced intensity. Stratification by number of Charlson comorbidities revealed a higher impact among those with no comorbidities (up to IRR = 2.71, 95% CI = 2.60–2.83 at month 4 after the infection) than among those with one comorbidity (up to IRR = 1.41, 95% CI = 1.29–1.54 at month 3) or more than one comorbidity (up to IRR = 1.38, 95% CI = 1.29–1.47 at month 3) (Supplementary Fig. 3, Additional File 1). Detailed estimates of the IRR, in each cohort and in the pooled analysis, are reported in Supplementary Tables 12–14 (Additional File 1). The results were quite homogeneous in the two regional cohorts. The two populations of SARS-CoV-2 infected individuals showed similar increases in rates during the PAP, both in intensity and duration (Fig. 3). Heterogeneity between cohorts was highest in the first six months of the PAP for those with severe or moderate acute COVID-19 (Supplementary Table 14, Additional File 1). Adjustment for history bias is described in Supplementary Tables 15 and 16 (Additional File 1). Overall, ageing of individuals was associated to a higher risk of outcome in the general populations of the two regions (about + 3% rate for a one-year increase, in both cohorts). Furthermore, the average level of provision of outpatient care in the period was also associated to a higher risk of outcome.

Adjusted incidence rates, calculated from the repeated measures mixed models, are reported in Table 3. These measures express the rates that would have been observed in the absence of history bias related to ageing of individuals and to levels of provision of outpatient care in different periods of the pandemic. Goodness of fit of repeated measures mixed models was judged as excellent based on calibration plots before and after diagnosis (Supplementary Fig. 3, Additional File 1), and on linear calibration lines (Supplementary Table 17, Additional File 1).

**Table 3:** Adjusted incidence rate of selected outpatient care services per time-point, by COVID-19 severity

	All subjects		Low or mild COVID-19 severity		Moderate COVID-19 severity		Severe COVID-19	
Time-point (pre- and post-infection)	Emilia-Romagna	Veneto	Emilia-Romagna	Veneto	Emilia-Romagna	Veneto	Emilia-Romagna	Veneto
Control period	3.471	2.262	2.433	1.788	5.992	4.197	6.438	4.875
2	5.538	4.015	3.558	2.508	11.809	11.917	11.644	13.405
3	6.845	4.362	3.880	2.371	12.931	10.274	17.612	20.305
4	6.883	4.828	3.528	2.270	12.958	11.877	19.167	24.975
5	6.778	4.588	3.369	2.063	11.537	12.340	20.398	24.355
6	6.079	3.891	3.410	2.228	10.500	9.537	16.096	15.991
7	6.317	3.869	3.403	2.224	11.662	9.076	16.788	15.695
8	5.740	3.551	3.261	1.952	10.351	8.305	14.547	15.235
9	5.107	2.928	3.070	1.844	8.317	6.524	12.827	10.710
10	4.341	2.564	2.975	1.750	6.429	5.586	9.576	8.317
11	4.323	2.734	2.744	1.880	7.071	6.314	10.067	7.894
12	4.266	2.582	2.725	1.755	7.303	5.200	9.640	8.332
13	4.126	2.361	2.676	1.651	7.078	4.609	9.168	7.418
14	4.237	2.439	2.805	1.740	7.390	4.972	9.016	7.045
15	3.899	2.359	2.427	1.661	6.749	4.018	9.117	7.914
16	3.735	2.211	2.351	1.582	6.988	4.074	8.329	6.884
17	3.331	2.146	2.316	1.612	5.635	3.935	6.777	6.007
18	3.682	2.177	2.509	1.613	6.023	4.470	7.851	5.816
19	3.717	2.190	2.540	1.582	7.009	4.673	7.165	5.985
20	3.717	2.150	2.410	1.624	7.078	4.107	7.840	5.656
21	3.645	2.168	2.453	1.627	6.094	4.303	7.841	5.682
22	3.560	1.833	2.422	1.443	6.460	3.231	7.276	4.614
23	3.732	1.933	2.551	1.541	6.499	3.134	7.692	4.884
24	3.453	2.140	2.375	1.584	5.979	4.177	7.052	5.903

Notes: Adjusted incidence rates, expressed as the number of outpatient care services per 1,000 individuals per day, are shown for the control period (CP) and for each month of the post-acute phase (PAP) after SARS-CoV-2 infection; adjusted incidence rates are the rates predicted by the repeated measures mixed model for a population with the same size and characteristics of the analysed one, assuming: no censoring; that every subject is at risk for all the CP and the PAP; no ageing of individuals; and level of provision of outcome always equal to the observed average in 2019 (last year before the pandemic)

## Discussion

The study relies on the analysis of administrative healthcare data related to about 50,000 individuals positive for SARS-CoV-2 in two Italian regions in the first year of the pandemic. The results, fairly consistent between the two cohorts, showed an increase in the occurrence of specific outpatient care services after infection, compared to the period before the infection, after adjusting for the natural ageing of individuals and for outpatient healthcare delivery over time due to lockdowns or other restriction measures. Results are also consistent with previously reported data from other countries and for different population groups [33–37]. The pre-post study design took into consideration the fact that patients could be already suffering from one or more chronic diseases and require outpatient care before the infection. The increase may be due both to worsening of previously existing conditions and new symptoms, but also to catching up with previously cancelled visits. However, the magnitude of the increase suggests a prominent role of new symptoms, especially in subjects with no known previous comorbidities. Indirect evidence for this is that a higher IRR was found in the cohort without comorbidities than in the ones with comorbidities, although the latter had a higher incidence rate. Moreover, the increase was more evident (up to a four-fold rate) and prolonged (up to 24 months, even if with decreasing intensity) for individuals who experienced severe acute COVID-19, but it was also observed in individuals who experienced low or mild severity COVID-19. The increase in outpatient care rates may be explained by persistent effects of the infection, such as immune dysregulation or microvascular changes [1, 16], as well as the impact of delayed access to care during the pandemic’s first wave may have contributed, as already observed by other authors [38].

### Implications for research

The results reported in this study showed that the long-term effects of COVID-19 may be present well after the first year after the infection. In addition, acute COVID-19 severity had a strong impact on the risk of increased use of outpatient care. These findings underline the importance of focusing research on the long-term effects of COVID-19, especially after the first year. This is particularly true for hospitalized patients, but also low-risk individuals with no pre-existing comorbidity and mild acute presentation may need investigation [25].

### Implications for healthcare organization

Based on the results of our study, the access to outpatient care after SARS-CoV-2 infection was higher, especially in the first months but also in the following up to 24 months after the infection, compared to the year before the diagnosis. The relevant healthcare resource consumption related to such outpatient care should therefore be considered when planning the allocation of resources. Moreover, there is need for long-term follow-up of COVID-19 patients, as its long-term effects can last well after the first year, especially for hospitalized subjects. The data reported in this study can also be useful to provide adequate information to patients with SARS-CoV-2 about the duration and intensity of long-term effect of COVID-19. It also provides substantial support to the benefit of vaccination campaigns, as there is good quality evidence that vaccination reduces long-term effects of COVID-19. Finally, the early identification of subjects at high risk of long-term effects of COVID-19 could allow a wider access to care through telemedicine and could improve clinical outcomes [39].

### Limitations

Some limitations apply to this study. Firstly, the use of healthcare administrative databases as the data sources implies that some imprecision in the definition of variables may have been present. In particular, due to the source of the data, some specific outcomes (e.g. autonomic dysfunction), were not specifically considered. Secondly, the occurrence of selected outpatient care was used as the outcome variable, but no data on diagnoses that led to outpatient care is available. Outpatient care could however be part of the follow-up of specific categories of patients, regardless of persistence of symptoms. It is therefore possible that a small overestimation of the incidence of outpatient care in the PAP was present. Similarly, to avoid overestimating the effect of COVID-19 on outpatient care in the PAP and worsening of previous diseases, the analysis focused on health care use and did not consider drug prescriptions. Another potential limitation is in the criteria for the definition of acute COVID-19 severity, that are based on information available only for hospitalized subjects. Finally, the present study is about individuals diagnosed with SARS-CoV-2 for the first time in an early phase of the pandemic when vaccination was not available. Further research is needed to assess whether the observed increase in outpatient care usage would be more or less intense in a vaccinated population or in subjects with reinfections.

## Conclusions

Patients with COVID-19 experienced an increased number of healthcare visits in the first months after the acute infection. This increase may still be present at two years after the infection, especially for patients who were hospitalized during the acute phase of COVID-19 due to severe presentation of the disease.

## Electronic supplementary material

Below is the link to the electronic supplementary material.

Supplementary Material 1

## Data Availability

The individual data supporting the findings of this study are not publicly available because of security measures to protect personal data of participants. Aggregated data are available from the corresponding author on reasonable request and with the written permission of the Emilia-Romagna Region and the Veneto Region.

## Abbreviations

AP: Acute phase
CI: Confidence interval
CP: Control period
E-R: Emilia-Romagna
HMPC: High migratory pressure countries
IRR: Incidence rate ratio
LMPC: Low migratory pressure countries
PAP: Post-acute phase
