# Supplementary material for: The incidence of outpatient care within 24 months from SARS-CoV-2 infection in the general population: a multicenter population-based cohort study
Source: BMC Infect Dis. 2025 Jan 30;25:142. doi: 10.1186/s12879-025-10526-0 (PMC11783830; doi:10.1186/s12879-025-10526-0)
Supplement: Supplementary file 1 — Supplementary Material 1 [file 12879_2025_10526_MOESM1_ESM.docx]

**Supplementary Table 1.** Characteristics of the analysed individuals, by COVID-19 severity

|  |  | **Low or mild**  **COVID-19 severity** | | | | **Moderate**  **COVID-19 severity** | | | | **Severe**  **COVID-19** | | | |
| --- | --- | --- | --- | --- | --- | --- | --- | --- | --- | --- | --- | --- | --- |
|  |  | **Emilia-Romagna**  **(N = 19,817)** | | **Veneto**  **(N = 18,976)** | | **Emilia-Romagna**  **(N = 2,925)** | | **Veneto**  **(N = 1,997)** | | **Emilia-Romagna**  **(N = 4,398)** | | **Veneto**  **(N = 1,903)** | |
|  |  | **n** | **%** |  |  | **n** | **%** |  |  | **n** | **%** |  |  |
| Sex | Male | 8,479 | 42.8% | 8,156 | 43.0% | 1,510 | 51.6% | 1,063 | 53.2% | 2,685 | 61.1% | 1,220 | 64.1% |
| Age at diagnosis | 18-39 | 5,677 | 28.6% | 5,571 | 29.4% | 190 | 6.5% | 95 | 4.8% | 125 | 2.8% | 44 | 2.3% |
|  | 40-49 | 3,897 | 19.7% | 3,477 | 18.3% | 322 | 11.0% | 210 | 10.5% | 395 | 9.0% | 143 | 7.5% |
|  | 50-59 | 4,136 | 20.9% | 4,003 | 21.1% | 517 | 17.7% | 407 | 20.4% | 843 | 19.2% | 401 | 21.1% |
|  | 60-69 | 2,141 | 10.8% | 1,891 | 10.0% | 508 | 17.4% | 366 | 18.3% | 1,051 | 23.9% | 485 | 25.5% |
|  | 70-79 | 1,442 | 7.3% | 1,295 | 6.8% | 633 | 21.6% | 385 | 19.3% | 1,097 | 24.9% | 423 | 22.2% |
|  | ≥80 | 2,524 | 12.7% | 2,728 | 14.4% | 755 | 25.8% | 535 | 26.8% | 887 | 20.2% | 407 | 21.4% |
| Citizenship | Italian | 17,134 | 86.5% | 16,413 | 86.5% | 2,716 | 92.9% | 1,855 | 92.9% | 4,148 | 94.3% | 1,816 | 95.4% |
|  | CHMP | 2,645 | 13.3% | 2,532 | 13.3% | 205 | 7.0% | 140 | 7.0% | 246 | 5.6% | 84 | 4.4% |
|  | CLMP | 38 | 0.2% | 31 | 0.2% | 4 | 0.1% | 2 | 0.1% | 4 | 0.0% | 3 | 0.2% |

Notes: HMPC = high migratory pressure countries; LMPC = low migratory pressure countries

**Supplementary Table 2.** Prevalence of comorbidities at the time of infection according to the Charlson comorbidity index

| **Comorbidity** | **All subjects** | | | |
| --- | --- | --- | --- | --- |
|  | **Emilia-Romagna**  **(N = 27,140)** | | **Veneto**  **(N = 22,876)** | |
|  | **n** | **%** | **n** | **%** |
| Myocardial infarction | 375 | 1.4% | 212 | 0.9% |
| Congestive heart failure | 933 | 3.4% | 773 | 3.4% |
| Peripheral vascular disease | 400 | 1.5% | 230 | 1.0% |
| Cerebrovascular disease | 1,341 | 4.9% | 870 | 3.8% |
| Dementia | 948 | 3.5% | 557 | 2.4% |
| Chronic pulmonary disease | 709 | 2.6% | 293 | 1.3% |
| Connective tissue disease - rheumatic disease | 115 | 0.4% | 65 | 0.3% |
| Peptic ulcer disease | 100 | 0.4% | 47 | 0.2% |
| Mild liver disease | 246 | 0.9% | 148 | 0.6% |
| Diabetes without complications | 1,008 | 3.7% | 606 | 2.6% |
| Diabetes with complications | 246 | 0.9% | 92 | 0.4% |
| Paraplegia and hemiplegia | 130 | 0.5% | 91 | 0.4% |
| Renal disease | 561 | 2.1% | 302 | 1.3% |
| Cancer | 1,016 | 3.7% | 713 | 3.1% |
| Moderate or severe liver disease | 39 | 0.1% | 27 | 0.1% |
| Metastatic carcinoma | 204 | 0.8% | 136 | 0.6% |
| AIDS/HIV | 10 | 0.0% | 124 | 0.5% |

Notes: Comorbidities were defined based on the criteria of the Enhanced ICD-9-CM Charlson Comorbidity Index (Quan et al., 2005) applied to hospitalizations occurred within five years before the time of SARS-CoV-2 infection.

**Supplementary Table 3.** Time-at-risk and causes of interruption of follow-up, by COVID-19 severity

|  | **Low or mild**  **COVID-19 severity** | | **Moderate**  **COVID-19 severity** | | **Severe**  **COVID-19** | |
| --- | --- | --- | --- | --- | --- | --- |
|  | **Emilia-Romagna**  **(N = 19,817)** | **Veneto**  **(N = 18,976)** | **Emilia-Romagna**  **(N = 2,925)** | **Veneto**  **(N = 1,997)** | **Emilia-Romagna**  **(N = 4,398)** | **Veneto**  **(N = 1,903)** |
| Days-at-risk in the CP (mean ± SD) | 328.3 ± 10.3 | 328.5 ± 8.8 | 325.6 ± 16.7 | 325.7 ± 17.3 | 326.7 ± 14.4 | 326.8 ± 13.1 |
| Days-at-risk in the PAP (mean ± SD) | 652.6 ± 115.5 | 638.8 ± 132.5 | 618.3 ± 173.0 | 612.4 ± 180.3 | 626.3 ± 159.9 | 612.7 ± 177.1 |
| Complete 18-months follow-up (n %) | 16,504 (83.2%) | 14,373 (75.7%) | 2,428 (83.0%) | 1,563 (78.3%) | 3,818 (86.7%) | 1,549 (81.4%) |
| Death (n %) | 1,051 (5.3%) | 1,188 (6.3%) | 303 (10.4%) | 250 (12.5%) | 382 (8.7%) | 224 (11.8%) |
| Moved residence outside E-R Region (n %) | 123 (0.6%) | 144 (0.8%) | 12 (0.4%) | 4 (0.2%) | 14 (0.3%) | 7 (0.4%) |
| Reinfection (n %) | 2,149 (10.8%) | 3,271 (17.2%) | 184 (6.3%) | 180 (9.0%) | 184 (4.2%) | 123 (6.5%) |

Notes: SD = standard deviation; CP = control period before the SARS-CoV-2 infection; PAP = post-acute phase after SARS-CoV-2 infection.

**Supplementary Table 4.** Incidence rate and monthly proportion of subjects with outpatient care services per time-point - all subjects in the Emilia-Romagna cohort

| **Time-point**  **(pre- and post-infection)** | **Number of selected outpatient care services** | **Time-at-risk (years)** | **Analyzed subjects** | **Analyzed records** | **Crude incidence rate (services per 1,000 individuals per day)** | **Monthly proportion of subjects with events** | **Average provision level of outpatient care ^1^** |
| --- | --- | --- | --- | --- | --- | --- | --- |
| Control period | 30,136 | 24,369.0 | 27,140 | 297,952 | 3.388 | 6.2% | 0.941 |
| 2 | 2,807 | 2,115.5 | 26,578 | 26,578 | 3.635 | 6.0% | 0.607 |
| 3 | 4,358 | 2,155.9 | 26,642 | 26,642 | 5.538 | 9.2% | 0.794 |
| 4 | 4,805 | 2,163.8 | 26,581 | 26,581 | 6.084 | 9.5% | 0.846 |
| 5 | 4,577 | 2,162.0 | 26,504 | 26,504 | 5.800 | 8.8% | 0.833 |
| 6 | 4,366 | 2,156.3 | 26,401 | 26,401 | 5.547 | 8.9% | 0.910 |
| 7 | 4,890 | 2,149.4 | 26,319 | 26,319 | 6.233 | 10.3% | 0.982 |
| 8 | 4,400 | 2,144.4 | 26,217 | 26,217 | 5.621 | 9.1% | 0.964 |
| 9 | 3,604 | 2,137.1 | 26,128 | 26,128 | 4.620 | 7.8% | 0.885 |
| 10 | 2,914 | 2,128.0 | 26,035 | 26,035 | 3.752 | 6.4% | 0.869 |
| 11 | 3,281 | 2,121.4 | 25,930 | 25,930 | 4.237 | 7.5% | 1.000 |
| 12 | 3,228 | 2,116.1 | 25,856 | 25,856 | 4.179 | 7.6% | 1.005 |
| 13 | 3,039 | 2,111.6 | 25,789 | 25,789 | 3.943 | 7.4% | 0.990 |
| 14 | 3,227 | 2,107.1 | 25,740 | 25,740 | 4.196 | 7.8% | 1.027 |
| 15 | 3,014 | 2,101.0 | 25,680 | 25,680 | 3.930 | 7.0% | 1.042 |
| 16 | 2,812 | 2,094.6 | 25,614 | 25,614 | 3.678 | 6.6% | 1.001 |
| 17 | 2,295 | 2,079.2 | 25,500 | 25,500 | 3.024 | 5.7% | 0.930 |
| 18 | 2,582 | 2,058.5 | 25,217 | 25,217 | 3.436 | 6.5% | 0.985 |
| 19 | 2,859 | 2,044.4 | 25,023 | 25,023 | 3.831 | 7.3% | 1.076 |
| 20 | 2,800 | 2,030.3 | 24,868 | 24,868 | 3.778 | 7.2% | 1.063 |
| 21 | 2,658 | 2,012.2 | 24,679 | 24,679 | 3.619 | 7.1% | 1.035 |
| 22 | 2,363 | 1,976.9 | 24,398 | 24,398 | 3.275 | 6.0% | 0.953 |
| 23 | 2,550 | 1,907.1 | 23,686 | 23,686 | 3.663 | 6.9% | 1.017 |
| 24 | 2,471 | 1,858.2 | 22,885 | 22,885 | 3.643 | 7.0% | 1.080 |

Notes: ^1^ = the provision level of outpatient care in a specific time-point is the observed daily average total number of outpatient care services in that time-point in the Local Health Unit of residence, normalized for its observed daily average in 2019 (last year before the pandemic).

**Supplementary Table 5.** Incidence rate and monthly proportion of subjects with events per time-point - subjects with low or mild COVID-19 severity in the Emilia-Romagna cohort

| **Time-point (pre- and post-infection)** | **Number of selected outpatient care services** | **Time-at-risk (years)** | **Analyzed subjects** | **Analyzed records** | **Crude incidence rate (services per 1,000 individuals per day)** | **Monthly proportion of subjects with events** | **Average provision level of outpatient care ^1^** |
| --- | --- | --- | --- | --- | --- | --- | --- |
| Control period | 15,506 | 17,823.6 | 19,817 | 217,658 | 2.383 | 4.7% | 0.929 |
| 2 | 1,501 | 1,607.9 | 19,761 | 19,761 | 2.558 | 4.6% | 0.654 |
| 3 | 1,949 | 1,604.7 | 19,658 | 19,658 | 3.328 | 6.1% | 0.823 |
| 4 | 1,878 | 1,601.4 | 19,593 | 19,593 | 3.213 | 5.8% | 0.851 |
| 5 | 1,742 | 1,597.7 | 19,530 | 19,530 | 2.987 | 5.5% | 0.846 |
| 6 | 1,874 | 1,592.2 | 19,460 | 19,460 | 3.225 | 5.9% | 0.930 |
| 7 | 2,015 | 1,588.3 | 19,405 | 19,405 | 3.476 | 6.5% | 0.985 |
| 8 | 1,881 | 1,584.2 | 19,346 | 19,346 | 3.253 | 5.8% | 0.960 |
| 9 | 1,633 | 1,579.2 | 19,284 | 19,284 | 2.833 | 5.3% | 0.884 |
| 10 | 1,547 | 1,572.9 | 19,215 | 19,215 | 2.695 | 4.9% | 0.898 |
| 11 | 1,604 | 1,568.7 | 19,158 | 19,158 | 2.801 | 5.4% | 1.009 |
| 12 | 1,559 | 1,565.7 | 19,108 | 19,108 | 2.728 | 5.4% | 1.000 |
| 13 | 1,502 | 1,562.2 | 19,061 | 19,061 | 2.634 | 5.3% | 0.996 |
| 14 | 1,636 | 1,558.6 | 19,018 | 19,018 | 2.876 | 5.7% | 1.032 |
| 15 | 1,433 | 1,554.6 | 18,983 | 18,983 | 2.525 | 5.1% | 1.047 |
| 16 | 1,328 | 1,550.1 | 18,939 | 18,939 | 2.347 | 4.7% | 0.996 |
| 17 | 1,211 | 1,536.6 | 18,845 | 18,845 | 2.159 | 4.4% | 0.932 |
| 18 | 1,367 | 1,518.9 | 18,602 | 18,602 | 2.466 | 4.9% | 1.005 |
| 19 | 1,517 | 1,507.2 | 18,439 | 18,439 | 2.757 | 5.5% | 1.082 |
| 20 | 1,396 | 1,496.0 | 18,311 | 18,311 | 2.557 | 5.3% | 1.066 |
| 21 | 1,366 | 1,480.7 | 18,159 | 18,159 | 2.527 | 5.1% | 1.030 |
| 22 | 1,238 | 1,450.7 | 17,915 | 17,915 | 2.338 | 4.6% | 0.971 |
| 23 | 1,350 | 1,395.2 | 17,325 | 17,325 | 2.651 | 5.4% | 1.036 |
| 24 | 1,282 | 1,356.3 | 16,707 | 16,707 | 2.590 | 5.2% | 1.079 |

Notes: ^1^ = the provision level of outpatient care in a specific time-point is the observed daily average total number of outpatient care services in that time-point in the Local Health Unit of residence, normalized for its observed daily average in 2019 (last year before the pandemic).

**Supplementary Table 6.** Incidence rate and monthly proportion of subjects with events per time-point - subjects with moderate COVID-19 severity in the Emilia-Romagna cohort

| **Time-point**  **(pre- and post-infection)** | **Number of selected outpatient care services** | **Time-at-risk (years)** | **Analyzed subjects** | **Analyzed records** | **Crude incidence rate (services per 1,000 individuals per day)** | **Monthly proportion of subjects with events** | **Average provision level of outpatient care ^1^** |
| --- | --- | --- | --- | --- | --- | --- | --- |
| Control period | 5,550 | 2,609.4 | 2,925 | 32,043 | 5.827 | 10.4% | 0.969 |
| 2 | 537 | 213.5 | 2,821 | 2,821 | 6.890 | 9.6% | 0.476 |
| 3 | 784 | 225.3 | 2,826 | 2,826 | 9.535 | 15.5% | 0.691 |
| 4 | 889 | 226.4 | 2,810 | 2,810 | 10.757 | 16.0% | 0.798 |
| 5 | 772 | 225.9 | 2,786 | 2,786 | 9.364 | 14.2% | 0.776 |
| 6 | 745 | 224.7 | 2,764 | 2,764 | 9.085 | 14.3% | 0.844 |
| 7 | 899 | 223.0 | 2,746 | 2,746 | 11.046 | 17.5% | 0.958 |
| 8 | 805 | 222.0 | 2,722 | 2,722 | 9.935 | 15.8% | 0.962 |
| 9 | 589 | 221.0 | 2,707 | 2,707 | 7.302 | 11.6% | 0.883 |
| 10 | 419 | 219.5 | 2,702 | 2,702 | 5.230 | 9.7% | 0.805 |
| 11 | 537 | 218.8 | 2,683 | 2,683 | 6.725 | 11.3% | 0.969 |
| 12 | 565 | 218.0 | 2,674 | 2,674 | 7.101 | 11.9% | 1.007 |
| 13 | 516 | 216.9 | 2,662 | 2,662 | 6.518 | 12.1% | 0.968 |
| 14 | 548 | 216.2 | 2,656 | 2,656 | 6.944 | 12.2% | 1.003 |
| 15 | 511 | 215.6 | 2,642 | 2,642 | 6.493 | 11.0% | 1.016 |
| 16 | 516 | 214.4 | 2,630 | 2,630 | 6.594 | 10.7% | 1.000 |
| 17 | 384 | 213.3 | 2,623 | 2,623 | 4.932 | 8.8% | 0.916 |
| 18 | 405 | 212.1 | 2,602 | 2,602 | 5.230 | 9.0% | 0.928 |
| 19 | 530 | 211.1 | 2,592 | 2,592 | 6.878 | 12.2% | 1.041 |
| 20 | 536 | 209.8 | 2,576 | 2,576 | 7.001 | 11.6% | 1.040 |
| 21 | 448 | 208.4 | 2,561 | 2,561 | 5.891 | 11.1% | 1.033 |
| 22 | 424 | 205.7 | 2,535 | 2,535 | 5.647 | 9.5% | 0.903 |
| 23 | 438 | 199.3 | 2,483 | 2,483 | 6.020 | 9.7% | 0.956 |
| 24 | 428 | 194.0 | 2,395 | 2,395 | 6.044 | 11.1% | 1.065 |

Notes: ^1^ = the provision level of outpatient care in a specific time-point is the observed daily average total number of outpatient care services in that time-point in the Local Health Unit of residence, normalized for its observed daily average in 2019 (last year before the pandemic).

**Supplementary Table 7.** Incidence rate and monthly proportion of subjects with events per time-point – subjects with severe COVID-19 in the Emilia-Romagna cohort

| **Time-point**  **(pre- and post-infection)** | **Number of selected outpatient care services** | **Time-at-risk (years)** | **Analyzed subjects** | **Analyzed records** | **Crude incidence rate (services per 1,000 individuals per day)** | **Monthly proportion of subjects with events** | **Average provision level of outpatient care ^1^** |
| --- | --- | --- | --- | --- | --- | --- | --- |
| Control period | 9,080 | 3,936.0 | 4,398 | 48,251 | 6.320 | 10.3% | 0.976 |
| 2 | 769 | 294.0 | 3,996 | 3,996 | 7.166 | 10.5% | 0.464 |
| 3 | 1,625 | 325.9 | 4,158 | 4,158 | 13.660 | 19.9% | 0.724 |
| 4 | 2,038 | 336.0 | 4,178 | 4,178 | 16.616 | 22.0% | 0.851 |
| 5 | 2,063 | 338.4 | 4,188 | 4,188 | 16.704 | 20.7% | 0.811 |
| 6 | 1,747 | 339.4 | 4,177 | 4,177 | 14.100 | 19.3% | 0.861 |
| 7 | 1,976 | 338.1 | 4,168 | 4,168 | 16.010 | 23.1% | 0.982 |
| 8 | 1,714 | 338.2 | 4,149 | 4,149 | 13.884 | 20.1% | 0.983 |
| 9 | 1,382 | 336.8 | 4,137 | 4,137 | 11.241 | 16.9% | 0.890 |
| 10 | 948 | 335.6 | 4,118 | 4,118 | 7.739 | 11.6% | 0.775 |
| 11 | 1,140 | 333.9 | 4,089 | 4,089 | 9.355 | 14.4% | 0.982 |
| 12 | 1,104 | 332.4 | 4,074 | 4,074 | 9.101 | 15.1% | 1.024 |
| 13 | 1,021 | 332.5 | 4,066 | 4,066 | 8.413 | 14.2% | 0.977 |
| 14 | 1,043 | 332.3 | 4,066 | 4,066 | 8.600 | 14.7% | 1.021 |
| 15 | 1,070 | 330.8 | 4,055 | 4,055 | 8.862 | 13.7% | 1.038 |
| 16 | 968 | 330.1 | 4,045 | 4,045 | 8.034 | 12.8% | 1.027 |
| 17 | 700 | 329.3 | 4,032 | 4,032 | 5.824 | 10.2% | 0.928 |
| 18 | 810 | 327.4 | 4,013 | 4,013 | 6.777 | 11.9% | 0.930 |
| 19 | 812 | 326.1 | 3,992 | 3,992 | 6.823 | 12.3% | 1.073 |
| 20 | 868 | 324.5 | 3,981 | 3,981 | 7.328 | 13.4% | 1.066 |
| 21 | 844 | 323.2 | 3,959 | 3,959 | 7.155 | 13.3% | 1.060 |
| 22 | 701 | 320.6 | 3,948 | 3,948 | 5.991 | 10.5% | 0.904 |
| 23 | 762 | 312.6 | 3,878 | 3,878 | 6.679 | 12.2% | 0.970 |
| 24 | 761 | 307.8 | 3,783 | 3,783 | 6.773 | 12.0% | 1.093 |

Notes: ^1^ = the provision level of outpatient care in a specific time-point is the observed daily average total number of outpatient care services in that time-point in the Local Health Unit of residence, normalized for its observed daily average in 2019 (last year before the pandemic).

**Supplementary Table 8.** Incidence rate and monthly proportion of subjects with outpatient care services per time-point - all subjects in the Veneto cohort

| **Time-point**  **(pre- and post-infection)** | **Number of selected outpatient care services** | **Time-at-risk (years)** | **Analyzed subjects** | **Analyzed records** | **Crude incidence rate (services per 1,000 individuals per day)** | **Monthly proportion of subjects with events** | **Average provision level of outpatient care ^1^** |
| --- | --- | --- | --- | --- | --- | --- | --- |
| Control period | 17,001 | 20,565.8 | 22,876 | 251,250 | 2.265 | 5.2% | 0.949 |
| 2 | 2,375 | 1,823.2 | 22,620 | 22,620 | 3.569 | 7.4% | 0.883 |
| 3 | 2,943 | 1,836.0 | 22,583 | 22,583 | 4.392 | 8.5% | 1.000 |
| 4 | 3,237 | 1,835.2 | 22,498 | 22,498 | 4.832 | 8.2% | 0.964 |
| 5 | 2,991 | 1,825.7 | 22,358 | 22,358 | 4.488 | 7.7% | 0.941 |
| 6 | 2,722 | 1,816.9 | 22,260 | 22,260 | 4.105 | 7.9% | 1.041 |
| 7 | 2,834 | 1,808.7 | 22,131 | 22,131 | 4.293 | 8.0% | 1.103 |
| 8 | 2,506 | 1,799.2 | 22,008 | 22,008 | 3.816 | 6.9% | 1.061 |
| 9 | 1,918 | 1,788.1 | 21,869 | 21,869 | 2.939 | 5.7% | 0.973 |
| 10 | 1,667 | 1,775.7 | 21,710 | 21,710 | 2.572 | 5.5% | 0.982 |
| 11 | 1,956 | 1,766.8 | 21,587 | 21,587 | 3.033 | 6.2% | 1.101 |
| 12 | 1,871 | 1,759.8 | 21,501 | 21,501 | 2.913 | 6.1% | 1.105 |
| 13 | 1,682 | 1,752.6 | 21,415 | 21,415 | 2.629 | 5.6% | 1.094 |
| 14 | 1,787 | 1,746.1 | 21,329 | 21,329 | 2.804 | 6.3% | 1.137 |
| 15 | 1,718 | 1,739.0 | 21,244 | 21,244 | 2.707 | 5.9% | 1.137 |
| 16 | 1,544 | 1,731.1 | 21,175 | 21,175 | 2.444 | 5.5% | 1.089 |
| 17 | 1,443 | 1,708.8 | 20,996 | 20,996 | 2.314 | 5.1% | 1.052 |
| 18 | 1,503 | 1,685.4 | 20,675 | 20,675 | 2.443 | 5.6% | 1.105 |
| 19 | 1,584 | 1,667.2 | 20,427 | 20,427 | 2.603 | 5.8% | 1.164 |
| 20 | 1,529 | 1,651.1 | 20,240 | 20,240 | 2.537 | 5.7% | 1.146 |
| 21 | 1,497 | 1,633.9 | 20,036 | 20,036 | 2.510 | 5.5% | 1.113 |
| 22 | 1,186 | 1,599.2 | 19,778 | 19,778 | 2.032 | 4.6% | 1.065 |
| 23 | 1,288 | 1,532.1 | 19,113 | 19,113 | 2.303 | 5.2% | 1.126 |
| 24 | 1,400 | 1,475.1 | 18,273 | 18,273 | 2.600 | 5.6% | 1.136 |

Notes: ^1^ = the provision level of outpatient care in a specific time-point is the observed daily average total number of outpatient care services in that time-point in the Local Health Unit of residence, normalized for its observed daily average in 2019 (last year before the pandemic).

**Supplementary Table 9.** Incidence rate and monthly proportion of subjects with events per time-point - subjects with low and mild COVID-19 severity in the Veneto cohort

| **Time-point (pre- and post-infection)** | **Number of selected outpatient care services** | **Time-at-risk (years)** | **Analyzed subjects** | **Analyzed records** | **Crude incidence rate (services per 1,000 individuals per day)** | **Monthly proportion of subjects with events** | **Average provision level of outpatient care ^1^** |
| --- | --- | --- | --- | --- | --- | --- | --- |
| Control period | 11,283 | 17,080.0 | 18,976 | 208,489 | 1.810 | 4.3% | 0.945 |
| 2 | 1,369 | 1,545.8 | 18,940 | 18,940 | 2.426 | 5.4% | 0.906 |
| 3 | 1,402 | 1,540.4 | 18,842 | 18,842 | 2.494 | 5.5% | 1.008 |
| 4 | 1,296 | 1,536.0 | 18,777 | 18,777 | 2.312 | 5.1% | 0.961 |
| 5 | 1,154 | 1,527.7 | 18,673 | 18,673 | 2.070 | 4.5% | 0.945 |
| 6 | 1,352 | 1,520.4 | 18,598 | 18,598 | 2.436 | 5.3% | 1.049 |
| 7 | 1,397 | 1,513.6 | 18,499 | 18,499 | 2.529 | 5.4% | 1.105 |
| 8 | 1,163 | 1,504.9 | 18,402 | 18,402 | 2.117 | 4.6% | 1.057 |
| 9 | 1,018 | 1,495.2 | 18,277 | 18,277 | 1.865 | 4.0% | 0.972 |
| 10 | 984 | 1,484.8 | 18,143 | 18,143 | 1.816 | 4.1% | 0.998 |
| 11 | 1,150 | 1,477.6 | 18,040 | 18,040 | 2.132 | 4.7% | 1.103 |
| 12 | 1,072 | 1,471.6 | 17,972 | 17,972 | 1.996 | 4.5% | 1.099 |
| 13 | 996 | 1,465.6 | 17,901 | 17,901 | 1.862 | 4.2% | 1.093 |
| 14 | 1,084 | 1,460.1 | 17,824 | 17,824 | 2.034 | 4.9% | 1.138 |
| 15 | 1,026 | 1,454.4 | 17,755 | 17,755 | 1.933 | 4.6% | 1.139 |
| 16 | 925 | 1,447.7 | 17,704 | 17,704 | 1.750 | 4.1% | 1.086 |
| 17 | 914 | 1,427.1 | 17,544 | 17,544 | 1.755 | 4.0% | 1.053 |
| 18 | 954 | 1,405.6 | 17,247 | 17,247 | 1.859 | 4.5% | 1.115 |
| 19 | 969 | 1,389.2 | 17,019 | 17,019 | 1.911 | 4.5% | 1.167 |
| 20 | 972 | 1,374.6 | 16,855 | 16,855 | 1.937 | 4.5% | 1.147 |
| 21 | 933 | 1,359.1 | 16,663 | 16,663 | 1.881 | 4.3% | 1.108 |
| 22 | 790 | 1,327.1 | 16,429 | 16,429 | 1.631 | 3.8% | 1.072 |
| 23 | 848 | 1,266.7 | 15,821 | 15,821 | 1.834 | 4.2% | 1.122 |
| 24 | 849 | 1,216.1 | 15,075 | 15,075 | 1.913 | 4.3% | 1.129 |

Notes: ^1^ = the provision level of outpatient care in a specific time-point is the observed daily average total number of outpatient care services in that time-point in the Local Health Unit of residence, normalized for its observed daily average in 2019 (last year before the pandemic).

**Supplementary Table 10.** Incidence rate and monthly proportion of subjects with events per time-point - subjects with moderate COVID-19 severity in the Veneto cohort

| **Time-point**  **(pre- and post-infection)** | **Number of selected outpatient care services** | **Time-at-risk (years)** | **Analyzed subjects** | **Analyzed records** | **Crude incidence rate (services per 1,000 individuals per day)** | **Monthly proportion of subjects with events** | **Average provision level of outpatient care ^1^** |
| --- | --- | --- | --- | --- | --- | --- | --- |
| Control period | 2,707 | 1,781.7 | 1,997 | 21,873 | 4.162 | 9.2% | 0.968 |
| 2 | 539 | 150.3 | 1,941 | 1,941 | 9.825 | 17.2% | 0.784 |
| 3 | 570 | 154.1 | 1,944 | 1,944 | 10.135 | 19.7% | 0.973 |
| 4 | 666 | 153.9 | 1,910 | 1,910 | 11.852 | 18.9% | 0.980 |
| 5 | 656 | 153.2 | 1,888 | 1,888 | 11.732 | 20.2% | 0.935 |
| 6 | 541 | 152.0 | 1,876 | 1,876 | 9.750 | 17.2% | 1.012 |
| 7 | 560 | 151.7 | 1,865 | 1,865 | 10.112 | 17.2% | 1.099 |
| 8 | 499 | 151.0 | 1,847 | 1,847 | 9.054 | 14.9% | 1.087 |
| 9 | 353 | 149.9 | 1,839 | 1,839 | 6.452 | 11.2% | 0.986 |
| 10 | 283 | 148.7 | 1,825 | 1,825 | 5.216 | 10.1% | 0.929 |
| 11 | 366 | 147.8 | 1,812 | 1,812 | 6.784 | 12.5% | 1.094 |
| 12 | 314 | 147.3 | 1,804 | 1,804 | 5.841 | 11.9% | 1.136 |
| 13 | 270 | 146.9 | 1,798 | 1,798 | 5.035 | 10.3% | 1.100 |
| 14 | 300 | 146.3 | 1,794 | 1,794 | 5.618 | 11.0% | 1.130 |
| 15 | 241 | 145.4 | 1,785 | 1,785 | 4.540 | 9.8% | 1.129 |
| 16 | 236 | 144.7 | 1,773 | 1,773 | 4.469 | 9.6% | 1.100 |
| 17 | 215 | 143.4 | 1,759 | 1,759 | 4.108 | 8.9% | 1.052 |
| 18 | 244 | 142.1 | 1,743 | 1,743 | 4.703 | 10.2% | 1.065 |
| 19 | 276 | 141.1 | 1,731 | 1,731 | 5.360 | 11.1% | 1.150 |
| 20 | 240 | 140.2 | 1,716 | 1,716 | 4.692 | 10.0% | 1.141 |
| 21 | 250 | 139.2 | 1,709 | 1,709 | 4.919 | 9.8% | 1.141 |
| 22 | 168 | 137.7 | 1,699 | 1,699 | 3.343 | 7.5% | 1.051 |
| 23 | 179 | 133.9 | 1,666 | 1,666 | 3.662 | 7.7% | 1.160 |
| 24 | 232 | 130.0 | 1,610 | 1,610 | 4.890 | 9.6% | 1.175 |

Notes: ^1^ = the provision level of outpatient care in a specific time-point is the observed daily average total number of outpatient care services in that time-point in the Local Health Unit of residence, normalized for its observed daily average in 2019 (last year before the pandemic).

**Supplementary Table 11.** Incidence rate and monthly proportion of subjects with events per time-point – subjects with severe COVID-19 in the Veneto cohort

| **Time-point**  **(pre- and post-infection)** | **Number of selected outpatient care services** | **Time-at-risk (years)** | **Analyzed subjects** | **Analyzed records** | **Crude incidence rate (services per 1,000 individuals per day)** | **Monthly proportion of subjects with events** | **Average provision level of outpatient care ^1^** |
| --- | --- | --- | --- | --- | --- | --- | --- |
| Control period | 3,011 | 1,704.0 | 1,903 | 20,888 | 4.841 | 10.3% | 0.975 |
| 2 | 467 | 127.2 | 1,739 | 1,739 | 10.061 | 17.5% | 0.736 |
| 3 | 971 | 141.4 | 1,797 | 1,797 | 18.808 | 28.3% | 0.954 |
| 4 | 1,275 | 145.2 | 1,811 | 1,811 | 24.050 | 28.7% | 0.978 |
| 5 | 1,181 | 144.8 | 1,797 | 1,797 | 22.342 | 27.4% | 0.911 |
| 6 | 829 | 144.5 | 1,786 | 1,786 | 15.720 | 24.6% | 0.985 |
| 7 | 877 | 143.4 | 1,767 | 1,767 | 16.753 | 24.8% | 1.087 |
| 8 | 844 | 143.2 | 1,759 | 1,759 | 16.146 | 22.2% | 1.071 |
| 9 | 547 | 143.0 | 1,753 | 1,753 | 10.476 | 17.0% | 0.963 |
| 10 | 400 | 142.2 | 1,742 | 1,742 | 7.704 | 14.8% | 0.875 |
| 11 | 440 | 141.4 | 1,735 | 1,735 | 8.522 | 15.0% | 1.081 |
| 12 | 485 | 140.9 | 1,725 | 1,725 | 9.432 | 16.4% | 1.140 |
| 13 | 416 | 140.1 | 1,716 | 1,716 | 8.138 | 14.4% | 1.099 |
| 14 | 403 | 139.6 | 1,711 | 1,711 | 7.908 | 15.0% | 1.132 |
| 15 | 451 | 139.2 | 1,704 | 1,704 | 8.877 | 15.6% | 1.131 |
| 16 | 383 | 138.6 | 1,698 | 1,698 | 7.568 | 14.8% | 1.113 |
| 17 | 314 | 138.3 | 1,693 | 1,693 | 6.221 | 12.4% | 1.035 |
| 18 | 305 | 137.6 | 1,685 | 1,685 | 6.072 | 11.8% | 1.041 |
| 19 | 339 | 137.0 | 1,677 | 1,677 | 6.779 | 13.5% | 1.145 |
| 20 | 317 | 136.3 | 1,669 | 1,669 | 6.373 | 13.2% | 1.140 |
| 21 | 314 | 135.6 | 1,664 | 1,664 | 6.344 | 12.7% | 1.138 |
| 22 | 228 | 134.4 | 1,650 | 1,650 | 4.648 | 9.6% | 1.010 |
| 23 | 261 | 131.5 | 1,626 | 1,626 | 5.437 | 11.9% | 1.133 |
| 24 | 319 | 129.0 | 1,588 | 1,588 | 6.773 | 13.5% | 1.168 |

Notes: ^1^ = the provision level of outpatient care in a specific time-point is the observed daily average total number of outpatient care services in that time-point in the Local Health Unit of residence, normalized for its observed daily average in 2019 (last year before the pandemic).

**Supplementary Table 12.** Incidence rate ratio of selected outpatient care services comparing pre- and post-infection periods, by COVID-19 severity in the Emilia-Romagna Region

| **Time-point**  **(month after SARS-CoV-2 infection)** | **All subjects**  **(N = 27,140)** | | | **Low or mild**  **COVID-19 severity**  **(N = 19,817)** | | | **Moderate**  **COVID-19 severity**  **(N = 2,925)** | | | **Severe**  **COVID-19**  **(N = 4,398)** | | |
| --- | --- | --- | --- | --- | --- | --- | --- | --- | --- | --- | --- | --- |
|  | **IRR** | **95% CI** | **P value** | **IRR** | **95% CI** | **P value** | **IRR** | **95% CI** | **P value** | **IRR** | **95% CI** | **P value** |
| 2 | 1.589 | 1.505-1.678 | 0.0000 | 1.455 | 1.360-1.558 | 0.0000 | 1.967 | 1.693-2.285 | 0.0000 | 1.804 | 1.607-2.025 | 0.0000 |
| 3 | 1.968 | 1.886-2.054 | 0.0000 | 1.591 | 1.502-1.686 | 0.0000 | 2.156 | 1.937-2.399 | 0.0000 | 2.732 | 2.522-2.960 | 0.0000 |
| 4 | 1.980 | 1.897-2.067 | 0.0000 | 1.448 | 1.365-1.536 | 0.0000 | 2.161 | 1.949-2.396 | 0.0000 | 2.975 | 2.760-3.206 | 0.0000 |
| 5 | 1.950 | 1.862-2.041 | 0.0000 | 1.382 | 1.301-1.469 | 0.0000 | 1.924 | 1.726-2.144 | 0.0000 | 3.165 | 2.910-3.443 | 0.0000 |
| 6 | 1.750 | 1.675-1.828 | 0.0000 | 1.401 | 1.320-1.486 | 0.0000 | 1.752 | 1.576-1.946 | 0.0000 | 2.499 | 2.305-2.709 | 0.0000 |
| 7 | 1.820 | 1.749-1.893 | 0.0000 | 1.399 | 1.323-1.479 | 0.0000 | 1.946 | 1.771-2.139 | 0.0000 | 2.607 | 2.434-2.793 | 0.0000 |
| 8 | 1.653 | 1.585-1.725 | 0.0000 | 1.340 | 1.263-1.422 | 0.0000 | 1.727 | 1.560-1.912 | 0.0000 | 2.259 | 2.097-2.434 | 0.0000 |
| 9 | 1.470 | 1.404-1.538 | 0.0000 | 1.260 | 1.185-1.340 | 0.0000 | 1.387 | 1.233-1.561 | 0.0000 | 1.991 | 1.835-2.161 | 0.0000 |
| 10 | 1.249 | 1.190-1.311 | 0.0000 | 1.221 | 1.145-1.302 | 0.0000 | 1.072 | 0.951-1.209 | 0.2541 | 1.486 | 1.353-1.633 | 0.0000 |
| 11 | 1.246 | 1.192-1.302 | 0.0000 | 1.129 | 1.064-1.197 | 0.0001 | 1.180 | 1.058-1.316 | 0.0030 | 1.564 | 1.438-1.701 | 0.0000 |
| 12 | 1.229 | 1.176-1.285 | 0.0000 | 1.120 | 1.055-1.190 | 0.0002 | 1.219 | 1.088-1.365 | 0.0006 | 1.497 | 1.379-1.626 | 0.0000 |
| 13 | 1.189 | 1.137-1.242 | 0.0000 | 1.100 | 1.036-1.169 | 0.0020 | 1.181 | 1.063-1.312 | 0.0019 | 1.424 | 1.311-1.546 | 0.0000 |
| 14 | 1.221 | 1.169-1.276 | 0.0000 | 1.154 | 1.087-1.225 | 0.0000 | 1.233 | 1.107-1.374 | 0.0001 | 1.401 | 1.292-1.518 | 0.0000 |
| 15 | 1.124 | 1.073-1.176 | 0.0000 | 0.999 | 0.940-1.061 | 0.9676 | 1.126 | 1.003-1.265 | 0.0443 | 1.416 | 1.298-1.545 | 0.0000 |
| 16 | 1.077 | 1.027-1.129 | 0.0024 | 0.967 | 0.906-1.032 | 0.3100 | 1.166 | 1.038-1.310 | 0.0094 | 1.294 | 1.183-1.415 | 0.0000 |
| 17 | 0.960 | 0.912-1.010 | 0.1137 | 0.953 | 0.891-1.019 | 0.1600 | 0.941 | 0.830-1.066 | 0.3373 | 1.053 | 0.953-1.163 | 0.3132 |
| 18 | 1.062 | 1.012-1.115 | 0.0139 | 1.035 | 0.971-1.104 | 0.2899 | 1.006 | 0.884-1.144 | 0.9316 | 1.220 | 1.114-1.335 | 0.0000 |
| 19 | 1.074 | 1.026-1.125 | 0.0023 | 1.050 | 0.988-1.117 | 0.1158 | 1.171 | 1.050-1.305 | 0.0045 | 1.114 | 1.017-1.220 | 0.0203 |
| 20 | 1.074 | 1.025-1.126 | 0.0026 | 0.997 | 0.935-1.062 | 0.9244 | 1.182 | 1.058-1.321 | 0.0032 | 1.219 | 1.116-1.331 | 0.0000 |
| 21 | 1.055 | 1.006-1.106 | 0.0281 | 1.016 | 0.953-1.084 | 0.6224 | 1.018 | 0.913-1.137 | 0.7436 | 1.220 | 1.113-1.337 | 0.0000 |
| 22 | 1.035 | 0.983-1.089 | 0.1878 | 1.011 | 0.945-1.081 | 0.7471 | 1.084 | 0.958-1.225 | 0.1997 | 1.135 | 1.023-1.260 | 0.0167 |
| 23 | 1.098 | 1.046-1.152 | 0.0001 | 1.077 | 1.011-1.147 | 0.0217 | 1.102 | 0.971-1.251 | 0.1314 | 1.217 | 1.108-1.336 | 0.0000 |
| 24 | 1.017 | 0.967-1.070 | 0.5135 | 1.003 | 0.936-1.075 | 0.9259 | 1.015 | 0.906-1.138 | 0.7938 | 1.118 | 1.014-1.232 | 0.0245 |

Notes: Incidence rate ratios (IRR) with 95% confidence interval (CI) are shown for each month of the post-acute phase after SARS-CoV-2 infection, compared to the pre-infection control period.

**Supplementary Table 13.** Incidence rate ratio of selected outpatient care services comparing pre- and post-infection periods, by COVID-19 severity in the Veneto Region

| **Time-point**  **(month after SARS-CoV-2 infection)** | **All subjects**  **(N = 22,866)** | | | **Low or mild**  **COVID-19 severity**  **(N = 18,822)** | | | **Moderate**  **COVID-19 severity**  **(N = 1,998)** | | | **Severe**  **COVID-19**  **(N = 1,903)** | | |
| --- | --- | --- | --- | --- | --- | --- | --- | --- | --- | --- | --- | --- |
|  | **IRR** | **95% CI** | **P value** | **IRR** | **95% CI** | **P value** | **IRR** | **95% CI** | **P value** | **IRR** | **95% CI** | **P value** |
| 2 | 1.775 | 1.683-1.872 | 0.0000 | 1.402 | 1.313-1.497 | 0.0000 | 2.839 | 2.506-3.216 | 0.0000 | 2.750 | 2.411-3.135 | 0.0000 |
| 3 | 1.928 | 1.834-2.028 | 0.0000 | 1.326 | 1.241-1.416 | 0.0000 | 2.448 | 2.192-2.733 | 0.0000 | 4.165 | 3.751-4.625 | 0.0000 |
| 4 | 2.135 | 2.021-2.254 | 0.0000 | 1.269 | 1.187-1.357 | 0.0000 | 2.830 | 2.494-3.210 | 0.0000 | 5.123 | 4.574-5.737 | 0.0000 |
| 5 | 2.028 | 1.917-2.147 | 0.0000 | 1.153 | 1.072-1.241 | 0.0001 | 2.940 | 2.616-3.303 | 0.0000 | 4.996 | 4.447-5.612 | 0.0000 |
| 6 | 1.720 | 1.632-1.813 | 0.0000 | 1.246 | 1.165-1.332 | 0.0000 | 2.272 | 2.005-2.574 | 0.0000 | 3.280 | 2.932-3.669 | 0.0000 |
| 7 | 1.710 | 1.621-1.805 | 0.0000 | 1.244 | 1.162-1.331 | 0.0000 | 2.162 | 1.896-2.466 | 0.0000 | 3.219 | 2.873-3.607 | 0.0000 |
| 8 | 1.570 | 1.480-1.665 | 0.0000 | 1.092 | 1.014-1.175 | 0.0194 | 1.979 | 1.710-2.290 | 0.0000 | 3.125 | 2.758-3.541 | 0.0000 |
| 9 | 1.294 | 1.216-1.377 | 0.0000 | 1.031 | 0.955-1.114 | 0.4341 | 1.554 | 1.320-1.830 | 0.0000 | 2.197 | 1.919-2.514 | 0.0000 |
| 10 | 1.133 | 1.067-1.204 | 0.0001 | 0.979 | 0.908-1.055 | 0.5724 | 1.331 | 1.135-1.561 | 0.0004 | 1.706 | 1.490-1.953 | 0.0000 |
| 11 | 1.209 | 1.140-1.282 | 0.0000 | 1.051 | 0.978-1.130 | 0.1767 | 1.504 | 1.297-1.745 | 0.0000 | 1.619 | 1.412-1.857 | 0.0000 |
| 12 | 1.141 | 1.076-1.210 | 0.0000 | 0.982 | 0.913-1.055 | 0.6126 | 1.239 | 1.068-1.436 | 0.0046 | 1.709 | 1.490-1.960 | 0.0000 |
| 13 | 1.044 | 0.982-1.109 | 0.1714 | 0.923 | 0.856-0.995 | 0.0374 | 1.098 | 0.942-1.279 | 0.2307 | 1.522 | 1.318-1.756 | 0.0000 |
| 14 | 1.078 | 1.019-1.141 | 0.0090 | 0.973 | 0.908-1.042 | 0.4318 | 1.185 | 1.022-1.374 | 0.0249 | 1.445 | 1.259-1.658 | 0.0000 |
| 15 | 1.043 | 0.983-1.106 | 0.1609 | 0.929 | 0.865-0.997 | 0.0412 | 0.957 | 0.822-1.115 | 0.5758 | 1.623 | 1.413-1.865 | 0.0000 |
| 16 | 0.978 | 0.920-1.038 | 0.4615 | 0.885 | 0.820-0.954 | 0.0015 | 0.971 | 0.832-1.132 | 0.7043 | 1.412 | 1.233-1.616 | 0.0000 |
| 17 | 0.949 | 0.890-1.011 | 0.1018 | 0.901 | 0.834-0.974 | 0.0089 | 0.937 | 0.793-1.108 | 0.4482 | 1.232 | 1.065-1.426 | 0.0050 |
| 18 | 0.962 | 0.906-1.022 | 0.2080 | 0.902 | 0.839-0.970 | 0.0056 | 1.065 | 0.916-1.238 | 0.4144 | 1.193 | 1.022-1.392 | 0.0250 |
| 19 | 0.968 | 0.911-1.029 | 0.2965 | 0.885 | 0.821-0.954 | 0.0014 | 1.113 | 0.957-1.295 | 0.1639 | 1.228 | 1.064-1.416 | 0.0048 |
| 20 | 0.951 | 0.894-1.011 | 0.1051 | 0.908 | 0.842-0.980 | 0.0125 | 0.978 | 0.833-1.149 | 0.7898 | 1.160 | 1.007-1.337 | 0.0399 |
| 21 | 0.958 | 0.901-1.020 | 0.1815 | 0.910 | 0.844-0.981 | 0.0137 | 1.025 | 0.870-1.209 | 0.7666 | 1.166 | 1.003-1.355 | 0.0459 |
| 22 | 0.811 | 0.758-0.867 | 0.0000 | 0.807 | 0.744-0.875 | 0.0000 | 0.770 | 0.645-0.918 | 0.0037 | 0.946 | 0.800-1.119 | 0.5182 |
| 23 | 0.854 | 0.801-0.912 | 0.0000 | 0.862 | 0.796-0.933 | 0.0002 | 0.747 | 0.622-0.896 | 0.0017 | 1.002 | 0.864-1.161 | 0.9823 |
| 24 | 0.946 | 0.886-1.010 | 0.0990 | 0.886 | 0.816-0.961 | 0.0037 | 0.995 | 0.837-1.183 | 0.9550 | 1.211 | 1.043-1.406 | 0.0122 |

Notes: Incidence rate ratios (IRR) with 95% confidence interval (CI) are shown for each month of the post-acute phase after SARS-CoV-2 infection, compared to the pre-infection control period.

**Supplementary Table 14.** Pooled incidence rate ratio of selected outpatient care services comparing pre- and post-infection periods, by COVID-19 severity

| **Time-point**  **(month after SARS-CoV-2 infection)** | **All subjects** | | | | **Low or mild**  **COVID-19 severity** | | | | **Moderate**  **COVID-19 severity** | | | | **Severe**  **COVID-19** | | | |
| --- | --- | --- | --- | --- | --- | --- | --- | --- | --- | --- | --- | --- | --- | --- | --- | --- |
|  | **IRR** | **95% CI** | **p** | **tau** | **IRR** | **95% CI** | **p** | **tau** | **IRR** | **95% CI** | **p** | **tau** | **IRR** | **95% CI** | **p** | **tau** |
| 2 | 1.683 | 1.563-1.813 | 0.0000 | 0.0457 | 1.432 | 1.366-1.501 | 0.0000 | 0.0000 | 2.377 | 1.846-3.059 | 0.0000 | 0.1681 | 2.226 | 1.664-2.979 | 0.0000 | 0.2005 |
| 3 | 1.954 | 1.891-2.018 | 0.0000 | 0.0000 | 1.456 | 1.281-1.655 | 0.0000 | 0.0869 | 2.295 | 2.103-2.504 | 0.0000 | 0.0297 | 3.366 | 2.515-4.506 | 0.0000 | 0.2049 |
| 4 | 2.049 | 1.948-2.155 | 0.0000 | 0.0268 | 1.360 | 1.240-1.490 | 0.0000 | 0.0579 | 2.461 | 2.044-2.963 | 0.0000 | 0.1206 | 3.891 | 2.672-5.668 | 0.0000 | 0.2669 |
| 5 | 1.982 | 1.913-2.054 | 0.0000 | 0.0004 | 1.267 | 1.117-1.438 | 0.0002 | 0.0842 | 2.376 | 1.773-3.186 | 0.0000 | 0.2036 | 3.964 | 2.891-5.435 | 0.0000 | 0.2218 |
| 6 | 1.738 | 1.681-1.798 | 0.0000 | 0.0000 | 1.321 | 1.214-1.438 | 0.0000 | 0.0517 | 1.986 | 1.660-2.377 | 0.0000 | 0.1156 | 2.847 | 2.360-3.435 | 0.0000 | 0.1261 |
| 7 | 1.775 | 1.702-1.850 | 0.0000 | 0.0186 | 1.322 | 1.217-1.436 | 0.0000 | 0.0507 | 2.017 | 1.869-2.178 | 0.0000 | 0.0015 | 2.867 | 2.482-3.312 | 0.0000 | 0.0930 |
| 8 | 1.624 | 1.569-1.681 | 0.0000 | 0.0004 | 1.212 | 1.051-1.398 | 0.0083 | 0.0973 | 1.806 | 1.661-1.963 | 0.0000 | 0.0010 | 2.635 | 2.106-3.297 | 0.0000 | 0.1530 |
| 9 | 1.385 | 1.268-1.513 | 0.0000 | 0.0576 | 1.142 | 0.991-1.316 | 0.0658 | 0.0960 | 1.443 | 1.311-1.587 | 0.0000 | 0.0003 | 2.045 | 1.907-2.194 | 0.0000 | 0.0018 |
| 10 | 1.195 | 1.116-1.279 | 0.0000 | 0.0404 | 1.095 | 0.938-1.279 | 0.2511 | 0.1062 | 1.179 | 1.018-1.365 | 0.0280 | 0.0789 | 1.563 | 1.432-1.706 | 0.0000 | 0.0285 |
| 11 | 1.232 | 1.189-1.276 | 0.0000 | 0.0000 | 1.094 | 1.042-1.150 | 0.0003 | 0.0131 | 1.318 | 1.115-1.557 | 0.0012 | 0.1009 | 1.578 | 1.469-1.696 | 0.0000 | 0.0000 |
| 12 | 1.191 | 1.132-1.252 | 0.0000 | 0.0252 | 1.050 | 0.956-1.153 | 0.3068 | 0.0583 | 1.226 | 1.121-1.342 | 0.0000 | 0.0000 | 1.552 | 1.443-1.669 | 0.0000 | 0.0117 |
| 13 | 1.118 | 1.022-1.223 | 0.0153 | 0.0591 | 1.011 | 0.897-1.141 | 0.8530 | 0.0797 | 1.154 | 1.058-1.258 | 0.0012 | 0.0000 | 1.447 | 1.348-1.554 | 0.0000 | 0.0000 |
| 14 | 1.150 | 1.056-1.253 | 0.0014 | 0.0562 | 1.060 | 0.941-1.194 | 0.3385 | 0.0794 | 1.216 | 1.115-1.327 | 0.0000 | 0.0000 | 1.411 | 1.316-1.513 | 0.0000 | 0.0000 |
| 15 | 1.087 | 1.034-1.144 | 0.0012 | 0.0251 | 0.966 | 0.917-1.016 | 0.1798 | 0.0158 | 1.053 | 0.943-1.177 | 0.3594 | 0.0432 | 1.476 | 1.363-1.598 | 0.0000 | 0.0201 |
| 16 | 1.030 | 0.964-1.100 | 0.3885 | 0.0390 | 0.927 | 0.871-0.987 | 0.0181 | 0.0276 | 1.077 | 0.949-1.223 | 0.2512 | 0.0614 | 1.328 | 1.232-1.430 | 0.0000 | 0.0022 |
| 17 | 0.955 | 0.918-0.994 | 0.0231 | 0.0000 | 0.928 | 0.882-0.976 | 0.0039 | 0.0016 | 0.937 | 0.848-1.036 | 0.2051 | 0.0000 | 1.122 | 1.007-1.249 | 0.0370 | 0.0487 |
| 18 | 1.014 | 0.948-1.084 | 0.6919 | 0.0398 | 0.971 | 0.891-1.059 | 0.5058 | 0.0516 | 1.033 | 0.937-1.139 | 0.5170 | 0.0000 | 1.213 | 1.122-1.311 | 0.0000 | 0.0000 |
| 19 | 1.022 | 0.954-1.096 | 0.5313 | 0.0420 | 0.961 | 0.853-1.082 | 0.5090 | 0.0782 | 1.148 | 1.051-1.253 | 0.0022 | 0.0000 | 1.143 | 1.058-1.234 | 0.0007 | 0.0014 |
| 20 | 1.012 | 0.932-1.099 | 0.7685 | 0.0526 | 0.954 | 0.901-1.011 | 0.1107 | 0.0227 | 1.087 | 0.949-1.245 | 0.2268 | 0.0700 | 1.204 | 1.117-1.298 | 0.0000 | 0.0000 |
| 21 | 1.008 | 0.947-1.073 | 0.8052 | 0.0356 | 0.962 | 0.898-1.031 | 0.2760 | 0.0351 | 1.017 | 0.929-1.115 | 0.7100 | 0.0000 | 1.203 | 1.112-1.300 | 0.0000 | 0.0000 |
| 22 | 0.914 | 0.776-1.075 | 0.2772 | 0.1136 | 0.900 | 0.780-1.038 | 0.1485 | 0.0963 | 0.915 | 0.718-1.166 | 0.4728 | 0.1575 | 1.056 | 0.936-1.190 | 0.3769 | 0.0556 |
| 23 | 0.960 | 0.819-1.126 | 0.6197 | 0.1111 | 0.962 | 0.851-1.088 | 0.5351 | 0.0813 | 0.908 | 0.699-1.179 | 0.4677 | 0.1715 | 1.113 | 0.988-1.253 | 0.0781 | 0.0619 |
| 24 | 0.976 | 0.938-1.017 | 0.2445 | 0.0029 | 0.933 | 0.872-0.997 | 0.0415 | 0.0307 | 0.990 | 0.901-1.089 | 0.8394 | 0.0000 | 1.129 | 1.041-1.224 | 0.0033 | 0.0004 |

Notes: Incidence rate ratios (IRR) with 95% confidence interval (CI) are shown for each month of the post-acute phase after SARS-CoV-2 infection, compared to the pre-infection control period.

**Supplementary Table 15**. Effect of ageing of individuals on the rate of outpatient care services

|  | **Emilia-Romagna** | | | **Veneto** | | |
| --- | --- | --- | --- | --- | --- | --- |
|  | **IRR** | **95% CI** | **p** | **IRR** | **95% CI** | **p** |
| All subjects | 1.031 | 1.03-1.032 | 0.0000 | 1.035 | 1.034-1.036 | 0.0000 |
| Low or mild severity | 1.028 | 1.026-1.029 | 0.0000 | 1.033 | 1.031-1.034 | 0.0000 |
| Moderate severity | 1.012 | 1.008-1.015 | 0.0000 | 1.005 | 1.002-1.009 | 0.0046 |
| Severe COVID-19 | 1.006 | 1.003-1.009 | 0.0000 | 1.003 | 1.000-1.007 | 0.0627 |

Notes: the effect is referred to a one year increase. IRR = incidence rate ratio; CI = confidence interval.

**Supplementary Table 16**. Effect of the average level of provision of outpatient care on the rate of outpatient care services

|  | **Emilia-Romagna** | | | **Veneto** | | |
| --- | --- | --- | --- | --- | --- | --- |
|  | **IRR** | **95% CI** | **p** | **IRR** | **95% CI** | **p** |
| All subjects | 1.093 | 1.086-1.099 | 0.0000 | 1.081 | 1.073-1.089 | 0.0000 |
| Low or mild severity | 1.109 | 1.101-1.117 | 0.0000 | 1.086 | 1.077-1.096 | 0.0000 |
| Moderate severity | 1.098 | 1.081-1.114 | 0.0000 | 1.103 | 1.082-1.123 | 0.0000 |
| Severe COVID-19 | 1.081 | 1.069-1.094 | 0.0000 | 1.087 | 1.068-1.106 | 0.0000 |

Notes: the effect is referred to a 0.1 increase in the normalized level of provision of outpatient care. IRR = incidence rate ratio; CI = confidence interval.

**Supplementary Table 17.** Linear calibration of predicted incidence rates

|  | **Emilia-Romagna** | | **Veneto** | |
| --- | --- | --- | --- | --- |
|  | **Intercept** | **Slope** | **Intercept** | **Slope** |
| All subjects | 0.01 | 0.98 | 0.00 | 1.00 |
| Low or mild COVID-19 severity | 0.00 | 1.01 | 0.00 | 1.04 |
| Moderate COVID-19 severity | 0.01 | 0.97 | 0.02 | 0.92 |
| Severe COVID-19 | 0.03 | 0.90 | 0.04 | 0.87 |

Notes: linear calibration reports the coefficients of a simple linear regression line considering the observed values as the dependent variable and the predicted values as the independent variable. For perfect linear calibration, intercept should be equal to 0 and slope to 1.

**Supplementary Figure 1.** Definition of the provision level of outpatient care in a specific time-point


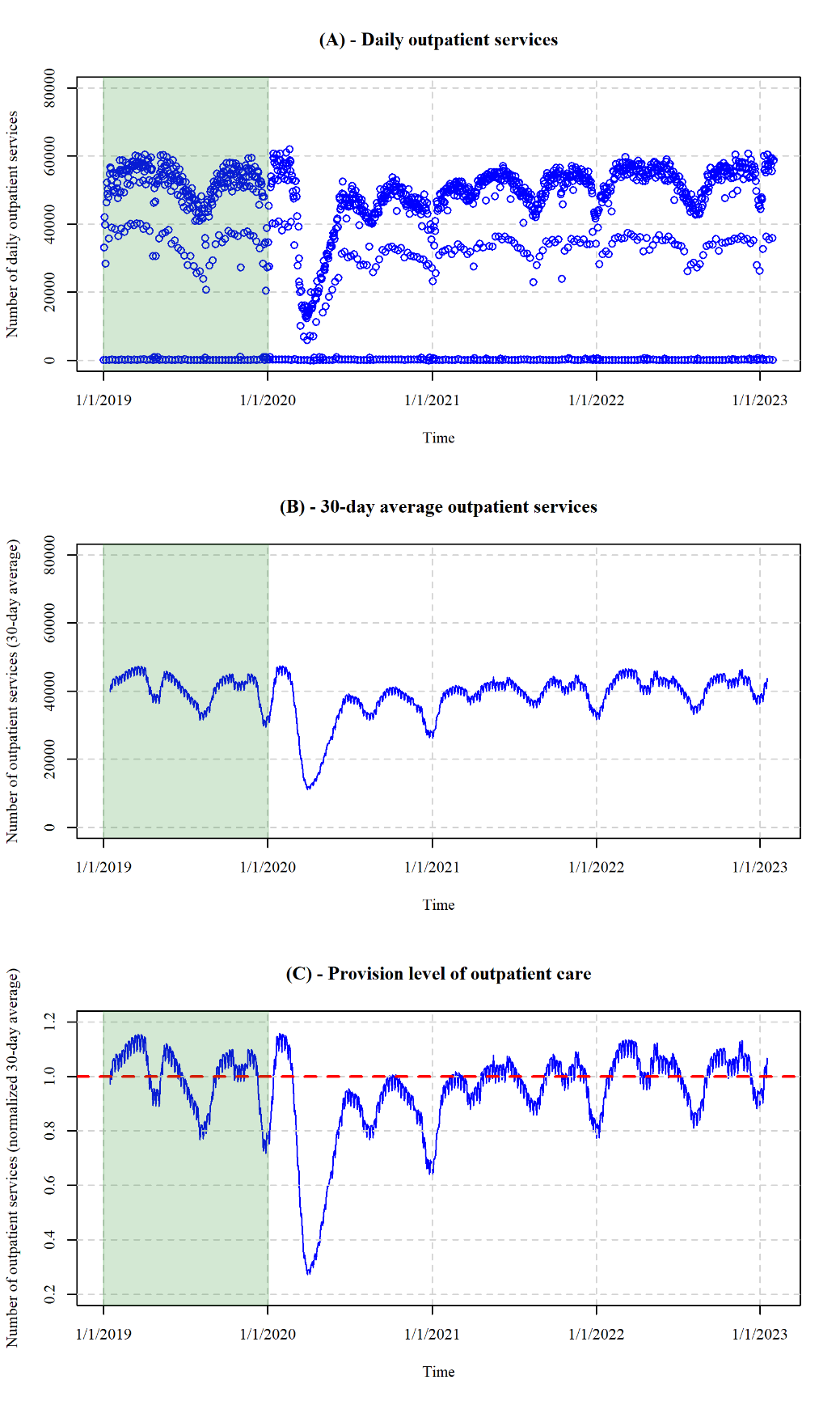


Notes: the provision level of outpatient care in a specific time-point (30-day period) is the observed daily average total number of outpatient care services in that time-point in the Local Health Unit of residence, normalized for its observed daily average in 2019 (last year before the pandemic). In subfigure (A), the observed daily total number of outpatient care services in one Local Health Unit in Emilia-Romagna is reported. In subfigure (B), the observed daily average total number of outpatient care services in 30-day periods around each calendar day for that Local Health Unit is reported. In subfigure (C), the provision level of outpatient care in 30-day periods around each calendar day, normalized for its observed daily average in 2019 (equal to 40,957.5 outpatient care services for that Local Health Unit), is reported.

**Supplementary Figure 2.** Observed incidence rates of selected outpatient care services before and after SARS-CoV-2 infection, by prevalence of Charlson comorbidities


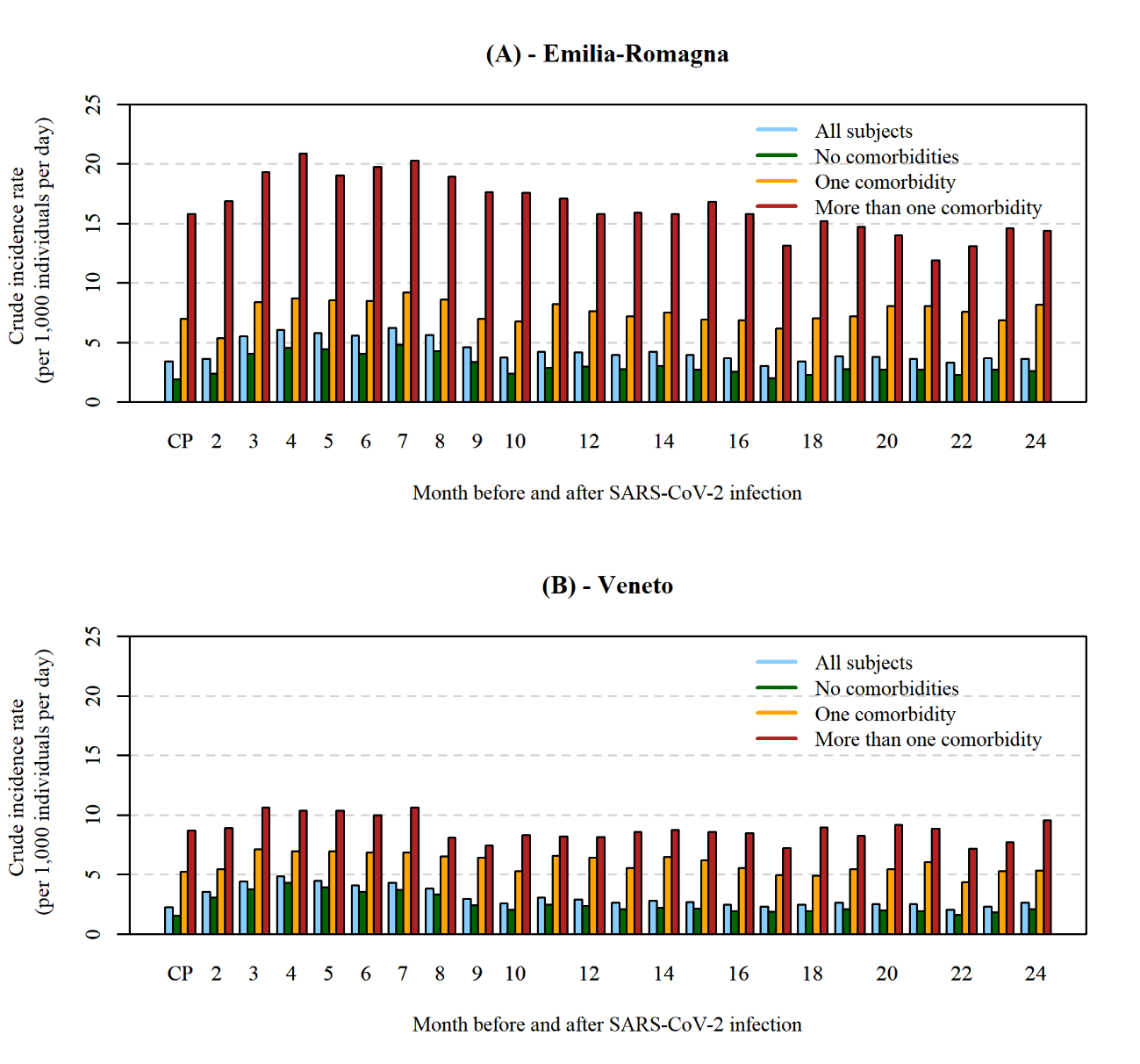


Notes: Observed crude incidence rates, expressed as the number of outpatient care services per 1,000 individuals per day, are shown for the control period (CP) and for each month of the post-acute phase after SARS-CoV-2 infection; (A) = Emilia-Romagna Region; (B) = Veneto Region; blue bars represent all analyzed subjects; green bars represent subjects with no comorbidities; yellow bars represent subjects with one comorbidity; red bars represent subjects with more than one comorbidity.

**Supplementary Figure 3.** Incidence rate ratio of selected outpatient care services comparing pre- and post-infection periods, by prevalence of Charlson comorbidities


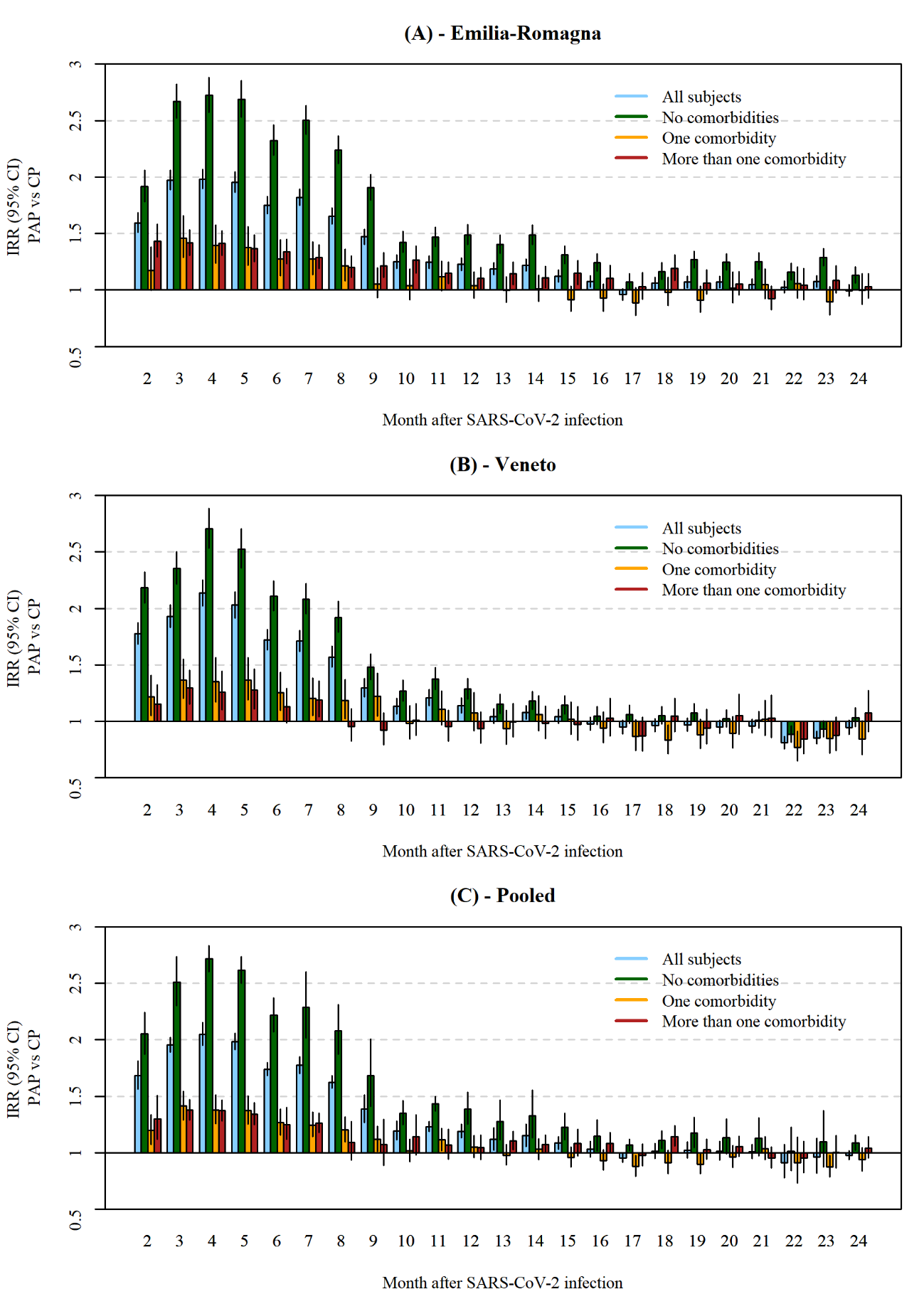


Notes: Incidence rate ratios (IRR) with 95% confidence intervals (CI) are shown for each month of the post-acute phase (PAP) after SARS-CoV-2 infection, compared to the pre-infection control period (CP); (A) = Emilia-Romagna Region; (B) = Veneto Region; (C) = Pooled results; blue bars represent all analyzed subjects; green bars represent subjects with no comorbidities; yellow bars represent subjects with one comorbidity; red bars represent subjects with more than one comorbidity; vertical error bars represent 95% CI.

**Supplementary Figure 4**. Calibration plot of observed and predicted incidence rates, per period before and after diagnosis


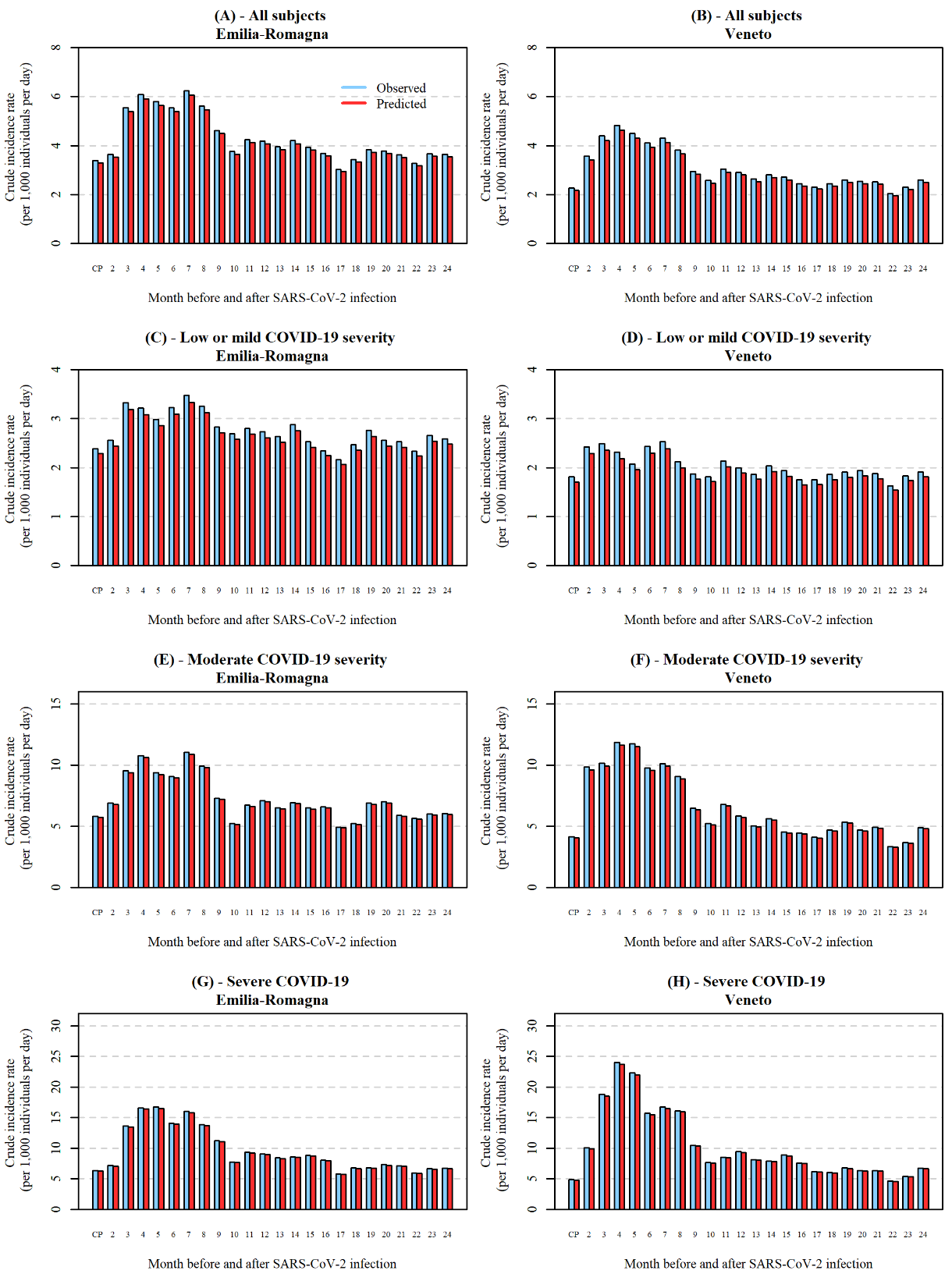


Notes: Observed and predicted crude incidence rates, expressed as the number of outpatient care services per 1,000 individuals per day, are shown for the control period (CP) and for each month of the post-acute phase after SARS-CoV-2 infection; (A) = All subjects in the Emilia-Romagna cohort; (B) = All subjects in the Veneto cohort; (C) = Subjects with low or mild COVID-19 severity in the Emilia-Romagna cohort; (D) = Subjects with low or mild COVID-19 severity in the Veneto cohort; blue bars represent all analyzed subjects; (E) = Subjects with moderate COVID-19 severity in the Emilia-Romagna cohort; (F) = Subjects with moderate COVID-19 severity in the Veneto cohort; (E) = Subjects with severe COVID-19 in the Emilia-Romagna cohort; (F) = Subjects with severe COVID-19 in the Veneto cohort. Blue bars represent observed crude incidence rates; red bars represent predicted incidence rates.

**Additional documentation.**

Additional documentation on the statistical analysis.

A generalized Poisson distribution was assumed for the outcome in mixed models, with dispersion parameter allowed to vary in each month of the PAP. A random intercept term was included to account for the different time-constant individual baseline risks of the outcome. Goodness of fit was assessed with internal calibration plots per month before and after diagnosis, and with the intercept and slope terms of the linear calibration regression line that considers the observed and predicted values of the outcomes.

Adjusted incidence rates were calculated as the rates predicted by the repeated measures mixed model for a population with the same size and characteristics of the analysed one, assuming: no censoring; that each subject was at risk for all the CP and the PAP; no ageing of individuals; and that the level of provision of the outcome was always equal to the observed average in 2019.

Pooling of IRRs obtained in the E-R and Veneto regions cohorts was carried out using random-effects meta-analysis with inverse variance weights and maximum likelihood estimator for between-study variance. Heterogeneity between cohorts was measured with the tau statistic.

Analyses were performed by E-R with SAS/STAT 15.1 (SAS Institute Inc., Cary, NC) and R 4.0.4 (The R Foundation for Statistical Computing, Wien) and by Veneto with SAS/STAT 13.1 statistical software.
